# Engineering *Streptomyces* sp. CPCC 204095 for the targeted high-level production of isatropolone A by elucidating its pathway-specific regulatory mechanism

**DOI:** 10.1186/s12934-024-02387-0

**Published:** 2024-04-16

**Authors:** Cong Zhang, Qianqian Xu, Jie Fu, Linzhuan Wu, Yihong Li, Yuan Lu, Yuanyuan Shi, Hongmin Sun, Xingxing Li, Lifei Wang, Bin Hong

**Affiliations:** CAMS Key Laboratory of Synthetic Biology for Drug Innovation, NHC Key Laboratory of Biotechnology for Microbial Drugs and State Key Laboratory of Bioactive Substances and Functions of Natural Medicines, Institute of Medicinal Biotechnology, Chinese Academy of Medical Sciences & Peking Union Medical College, Beijing, 100050 China

**Keywords:** Tropolone, Isatropolone, Pathway-specific regulators, Regulatory cascade, Cytochrome P450 monooxygenase, High-yield strain

## Abstract

**Background:**

Isatropolone A and C, produced by *Streptomyces* sp. CPCC 204095, belong to an unusual class of non-benzenoid aromatic compounds and contain a rare seven-membered ring structure. Isatropolone A exhibits potent activity against *Leishmania donovani*, comparable to the only oral drug miltefosine. However, its variably low productivity represents a limitation for this lead compound in the future development of new anti-leishmaniasis drugs to meet unmet clinical needs.

**Results:**

Here we first elucidated the regulatory cascade of biosynthesis of isatropolones, which consists of two SARP family regulators, IsaF and IsaJ. Through a series of in vivo and in vitro experiments, IsaF was identified as a pathway-specific activator that orchestrates the transcription of the gene cluster essential for isatropolone biosynthesis. Interestingly, IsaJ was found to only upregulate the expression of the cytochrome P450 monooxygenase IsaS, which is crucial for the yield and proportion of isatropolone A and C. Through targeted gene deletions of *isaJ* or *isaS*, we effectively impeded the conversion of isatropolone A to C. Concurrently, the facilitation of *isaF* overexpression governed by selected promoters, prompted the comprehensive activation of the production of isatropolone A. Furthermore, meticulous optimization of the fermentation parameters was conducted. These strategies culminated in the attainment of an unprecedented maximum yield—980.8 mg/L of isatropolone A—achieved in small-scale solid-state fermentation utilizing the genetically modified strains, thereby establishing the highest reported titer to date.

**Conclusion:**

In *Streptomyces* sp. CPCC 204095, the production of isatropolone A and C is modulated by the SARP regulators IsaF and IsaJ. IsaF serves as a master pathway-specific regulator for the production of isatropolones. IsaJ, on the other hand, only dictates the transcription of IsaS, the enzyme responsible for the conversion of isatropolone A and C. By engineering the expression of these pivotal genes, we have devised a strategy for genetic modification aimed at the selective and high-yield biosynthesis of isatropolone A. This study not only unveils the unique regulatory mechanisms governing isatropolone biosynthesis for the first time, but also establishes an essential engineering framework for the targeted high-level production of isatropolone A.

**Supplementary Information:**

The online version contains supplementary material available at 10.1186/s12934-024-02387-0.

## Background

**Fig. 1:**
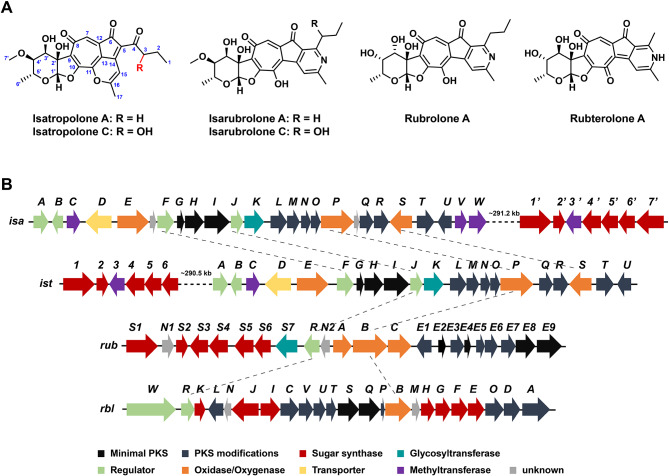
Chemical structures and biosynthesis gene clusters of a group of unique natural glycosylated tropolonoids produced in actinomycetes. (**A**) Chemical structures of isatropolones, isarubrolones, rubrolone, and rubterolone. (**B**) Schematic representation of the biosynthesis gene clusters of isatropolone in *Streptomyces* sp. CPCC 204095 (*isa*), isatropolone in *Streptomyces* Gö66 (*ist*), rubrolone in *Streptomyces* sp. KIB-H033 (*rub*) and rubterolone in *Actinomadura* sp. 5-2 (*rbl*). Grey dashed lines connect homologous genes of different gene clusters

Tropolones, found in the secondary metabolites of plants, fungi, and bacteria, is an important class of non-benzenoid aromatic compounds containing a typical seven-membered ring structure with a variety of biological properties such as antimicrobial, antiviral, and antitumor activities [1]. In bacteria, tropolones may be biosynthesized through the shikimate pathway and phenylacetic acid metabolism in, e.g., *Escherichia coli* [2], *Pseudomonas* [3–5], and *Streptomyces* [6]. Isatropolones, isarubrolones, rubrolones, and rubterolones (Fig. 1A) are a group of unique natural glycosylated tropolonoids produced in actinomycetes, sharing similar aglycone skeletons of a cyclopentanone ring, a tropolone unit, and a deoxysugar connected from C-2’ to the aglycone through a unique C-C bond. Yan et al. reported the biosynthetic gene cluster *rub* of rubrolones in *Streptomyces* sp. KIB-H033 and confirmed that the aglycone of rubrolones is co-catalyzed by type II polyketide synthase (PKS) followed by complex oxidative rearrangements through heterologous expression and gene knockout experiments [7]. Subsequently, Cai et al. identified the biosynthetic pathway of isatropolones in *Streptomyces* Gö66 and expressed *istG-R* of gene cluster *ist* of isatropolones in *S. lividans*, which can heterologously synthesize the aglycone moiety of isatropolones [8]. Guo et al. isolated several novel tropolone alkaloids, known as rubterolones, from *Actinomadura* sp. 5-2 which were found in the gut of the fungus-growing termite *Macrotermes natalensis*. Additionally, they proposed a biosynthesis pathway for rubterolones [9]. Bioinformatics comparative analysis of the biosynthetic gene cluster *ist* of isatropolones [8], *rub* of rubrolones [7] and *rbl* of rubterolones [9], shows that their core type II PKS genes and oxygenase genes have high similarity (Fig. 1B). While previous studies have provided a basic understanding of the biosynthesis of these glycosylated tropolonoids, the regulatory mechanism of their biosynthesis remains unclear.

In our previous work, we found that *Streptomyces* sp. CPCC 204095, isolated from the Miyun District of Beijing, China, capable of producing isatropolone A and C [10]. We sequenced the genome of CPCC 204095 and predicted secondary metabolism-related clusters using antiSMASH (version 5.2.0). A type II PKS biosynthetic gene cluster, named *isa* cluster, was supposed to be responsible for the production of isatropolones in CPCC 204095 [11], showing high similarity to the core genes of type II PKS genes of those in the gene clusters *ist, rub, rbl* (Fig. 1B, Additional file 1: Table S3). The chemical structures of isatropolone A and C are almost the same, except for the C-3 hydroxylation of the butyryl moiety in aglycone of isatropolone C (Fig. 1A). Isatropolones exhibit significant inhibitory activity against *Leishmania donovani* [8], the pathogen responsible for zoonotic leishmaniasis, which the WHO has described as a neglected tropical disease (http://www.who.int/leishmaniasis/en/). Skin and mucosal leishmaniasis can lead to permanent scars or severe disabilities, while visceral leishmaniasis can have a mortality rate of over 95% if not intervened in a timely manner. The inhibitory activity of isatropolone A (IC_50_ = 0.5 µM) against *L. donovani* is equivalent to that of miltefosine (IC_50_ = 0.3 µM), the only oral drug approved for the treatment of leishmaniasis [8]. The side effects (gastrointestinal toxicity, hemolytic side effects, damage to reproductive capacity, etc.) and high price of miltefosine limit its clinical use [12]. Isatropolone A showed four times more potent inhibition of *L. donovani* than isatropolone C, and even more potent than their corresponding non-enzymatically formed pyridine derivatives isarubrolones (Fig. 1A) [8]. Thus, isatropolone A represents a potential lead compound for the future development of novel anti-leishmaniasis drugs to fulfill the unmet clinical need.

Isatropolone A and C are produced by *Streptomyces* sp. CPCC 204095 at the early stage of fermentation (usually within 48 h) [13]. Usually, isatropolone C is the main product and the proportion of isatropolone A largely varies under different fermentation conditions. At the later stage of fermentation, corresponding pyridine derivatives of isatropolone A and C are formed with amines and various amine-containing compounds [8, 10]. The full chemical synthesis of these tropolones is challenging due to their unique structure of non-benzenoid aromatic seven-membered ring structure and the attachment of deoxysugar to the aglycone. Therefore, bioengineering approaches are almost the only feasible way to obtain strains with high yield of isatropolone A specifically without producing isatropolone C.

To accomplish this goal, in this work, we first characterized the regulatory role of putative pathway-specific transcriptional regulators in the biosynthesis of isatropolones through a series of in vivo and in vitro experiments. It is intriguing to find that the regulatory cascade of biosynthesis of isatropolones, composed of two SARP family regulators of IsaF and IsaJ, not only regulates the production of isatropolones but also controls the transformation of isatropolone A to isatropolone C. Disrupting the *isaJ* gene resulted in a shift of the main product from isatropolone C to A. Transcription analysis of the *isaJ* knockout strain indicated that the specifically decreased transcript level of *isaS*, a cytochrome P450 monooxygenase gene, led to the accumulation of isatropolone A, and indeed, the *isaS* knockout strain only produced isatropolone A. To further enhance the yield of isatropolone A, we developed engineered high-yield strains based on the *isaJ* and *isaS* knockout strains. Ultimately, we achieved nearly a gram per liter level of isatropolone A production in small-scale fermentation by optimizing the promoter and culture medium.

## Results and discussion

### Multiple transcriptional regulators in isatropolone biosynthetic gene cluster

Although previous studies have generally elucidated the biosynthetic mechanism of isatropolones, the regulatory mechanism remains uncharacterized. In the detailed analysis of the *isa* gene cluster, several transcriptional regulatory genes (*isaA, isaB, isaF* and *isaJ*) were predicted, suggesting a relatively complex regulatory mechanism for the biosynthesis of isatropolones (Fig. 1B). Among the 4 putative *isa* regulatory genes, only *isaJ* is conserved in four gene clusters (*isa, ist, rub* and *rbl*) of this group of tropolones produced in actinomycetes, indicating its crucial role in regulating the biosynthesis of these compounds. Bioinformatics analysis of IsaJ (263 amino acids) indicates that it belongs to the growing family of pathway-specific activators known as SARPs (*Streptomyces* antibiotic regulatory proteins) [14] and shows high similarity to RubR of *Streptomyces* sp. KIB-H003 (55% sequence identity and 71% sequence similarity) [7] and other SARPs such as PapR1 [15] of *S. pristinaespiralis* and OtcR [16] of *S. rimosus* which regulate antibiotic production of pristinamycin and terramycin respectively (Additional file 1: Fig. S1A). IsaF (296 amino acids), only present in *isa* and *ist* clusters, displays end-to-end similarity (∼ 40% sequence identity) to PapR1/2 and OtcR from *S. pristinaespiralis* and *S. rimosus* (Additional file 1: Fig. S1B). Analysis of the amino acid sequences of IsaJ and IsaF revealed an N-terminal of OmpR-like DNA binding domain and a C-terminal bacterial transcription activation domain, which was consistent with the structure of typical SARP family proteins (Additional file 1: Fig. S1). The *isaA* and *isaB* genes are in the proximity to the cluster border (Fig. 1B) and encode MerR and TetR family regulatory proteins, respectively. The deduced protein of IsaA is similar to TipAL-AS [17] of *S. lividans* with a typical N-terminal of MerR-like HTH DNA binding domain and a C-terminal domain of the TipAS family (Additional file 1: Fig. S2) which is activated by numerous cyclic thiopeptide antibiotics and leads to multidrug-resistance. IsaB containing a HTH DNA binding domain of TetR family at N-terminal (Additional file 1: Fig. S3A), shows high similarity to WP_189541946.1 of *S. gelaticus* and WP_206960553.1 of *S. beijiangensis* (Additional file 1: Fig. S3B), although bioactivities of these proteins have not been reported.

**Fig. 2:**
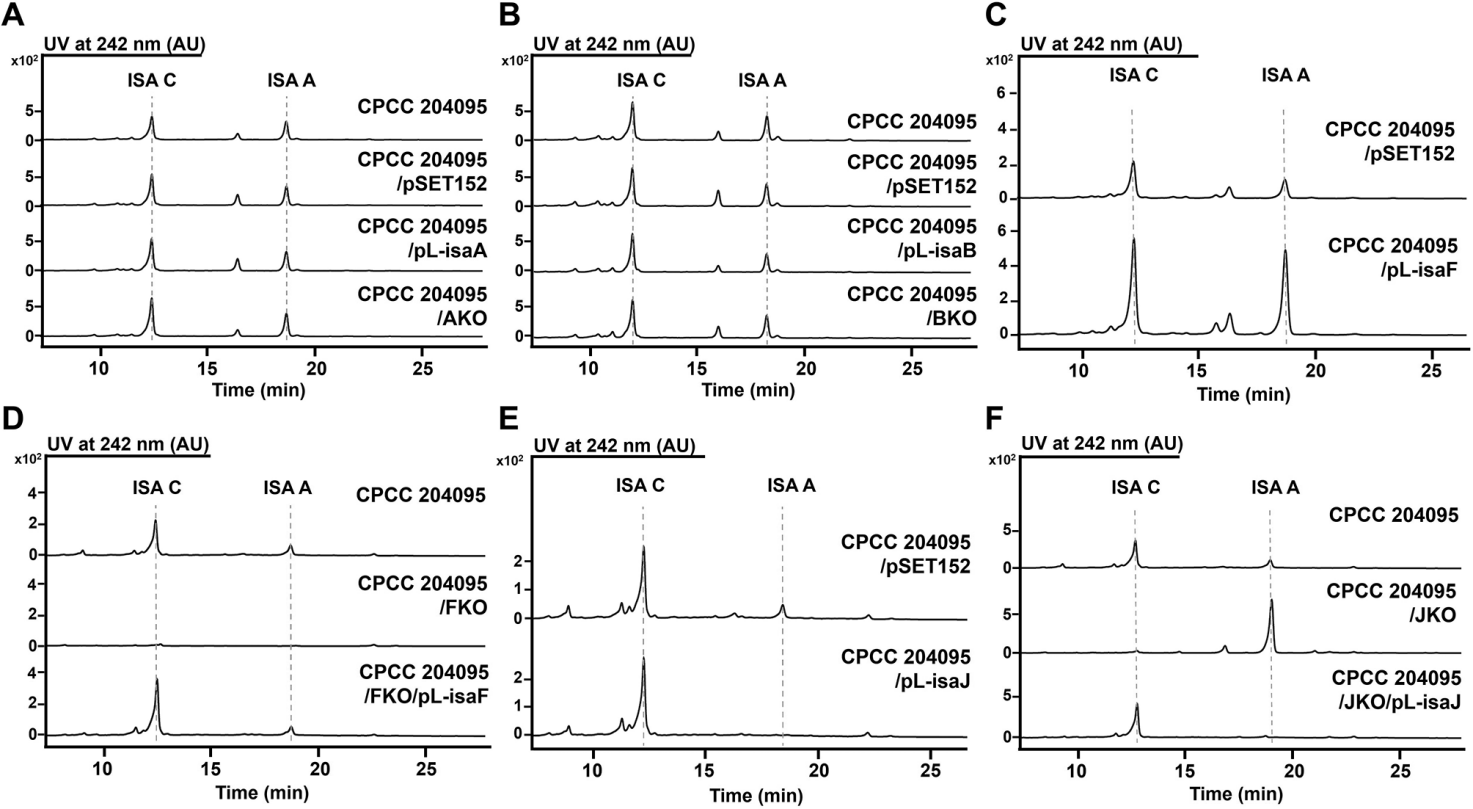
The impact of multiple transcription factors in the isatropolone biosynthetic gene cluster on the production of isatropolones. High-performance liquid chromatography (HPLC) analysis was used to monitor the production of isatropolone A (ISA A) and isatropolone C (ISA C) in the wild type strain (CPCC 204095), the empty vector control strain (CPCC 204095/pSET152), the overexpression strains (CPCC 204095/pL-isaA, CPCC 204095/pL-isaB, CPCC 204095/pL-isaF, CPCC 204095/pL-isaJ), gene knockout strains (AKO, BKO, FKO, and JKO), and genetic complementary strains (FKO/pL-isaF, JKO/pL-isaJ).

To investigate the regulatory function of four potential transcriptional regulators, their overexpression strains and knockout strains were constructed (Additional file 1: Fig. S4 and S5). Somewhat unexpectedly, the production level of isatropolones in both *isaA* and *isaB* overexpression and knockout strains showed no significant changes compared with CPCC 204095, suggesting that *isaA* and *isaB* may not be essential regulatory genes for isatropolone biosynthesis, at least under the current culture conditions (Fig. 2A and B). In the *isaF* overexpression strain (CPCC 204095/pL-isaF), the yield of both isatropolone A and C was approximately triple of that of the CPCC 204095 (Fig. 2C). Conversely, in the *isaF* knockout strain FKO, the deletion of *isaF* led to the abolishment of all isatropolone production, and the production was restored by reintroducing the *isaF* gene under the ermE*p promoter into FKO (Fig. 2D). These results demonstrated that IsaF is a pivotal positive regulator of isatropolones biosynthesis and suggested that IsaF might control the expression of multiple genes responsible for the biosynthesis of isatropolones. Surprisingly, the presence of an extra copy of *isaJ* under ermE*p resulted in a significant decrease in the production of isatropolone A in the CPCC 204095 (Fig. 2E). High-performance liquid chromatography (HPLC) - Mass spectrometry (MS) analysis of the fermentation products showed that isatropolones were produced in *isaJ* knockout strain, but the main product of JKO was isatropolone A while isatropolone C significantly decreased (Fig. 2F). Consistently, the production of isatropolone C was restored after the introduction of *isaJ* into JKO, while almost no isatropolone A was detected in the fermentation products (Fig. 2F). These results indicated that IsaJ does not affect the overall yield of isatropolones, but it does regulate the conversion of two key products, isatropolone A and isatropolone C.

The regulation of secondary metabolite production has been extensively studied and involves multiple levels of intertwined regulation in response to physiological and environmental condition alterations [18, 19]. Generally, the ultimate regulator of antibiotic production is a pathway-specific transcriptional regulator located in the biosynthetic cluster of secondary metabolites, controlling the production of the corresponding secondary metabolite. However, some gene clusters contain additional regulators to coordinate secondary metabolism with wider cell physiology and morphological development. Our preliminary results from knockout and overexpression of four putative regulators indicate that both *isaF* and *isaJ* are pathway-specific regulatory genes, but their regulatory mechanisms are entirely distinct, particularly in their specific effects on the production of isatropolone A and C, which warrant further exploration. While *isaA* and *isaB* do not currently impact the yield of isatropolones, it cannot be ruled out that they may be activated under specific physiological and environmental conditions.

### IsaF is a pathway-specific master regulator for biosynthesis of isatropolone

**Fig. 3:**
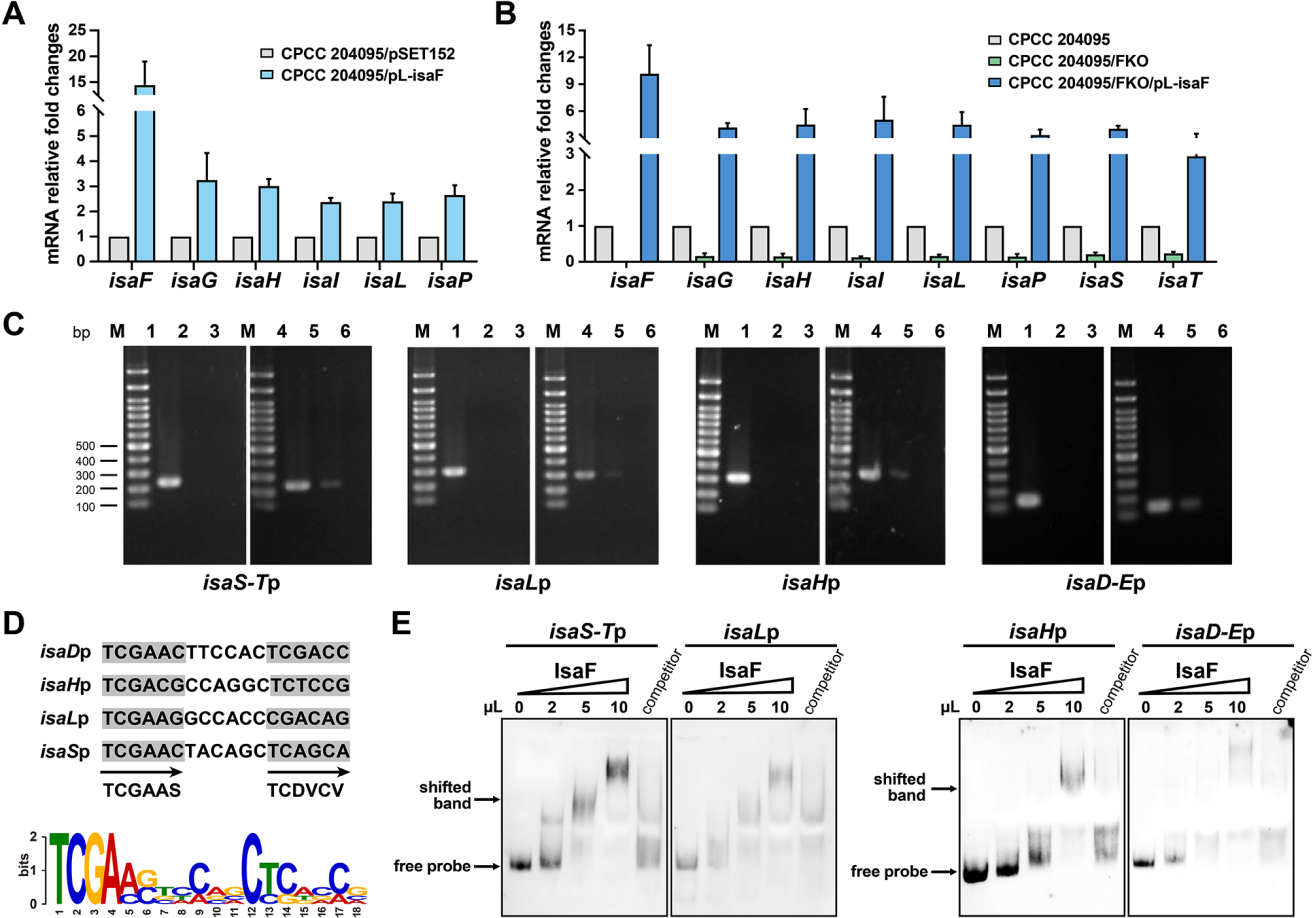
Identification of the target genes regulated by IsaF. (**A**, **B**) Transcriptional analysis of *isa* genes in the *isaF* overexpression strain (CPCC 204095/pL-isaF), and *isaF* knockout strain (FKO) and genetic complementary strain (FKO/pL-isaF) by RT-qPCR analysis. The relative mRNA level of the target genes was normalized to the principal sigma factor gene *hrdB*. The relative expression level of each sample was represented as the value related to the control strain CPCC 204095/pSET152 or CPCC 204095. Values are presented as mean ± SEM (two biological repeats for each strain). (**C**) Chromatin immunoprecipitation (ChIP) -PCR analysis of Flag-tagged IsaF. PCR using primers flanking target promoter regions was performed with immunoprecipitated DNA from CPCC 204095/FKO/pL-isaF-Flag (lanes 4–6) and negative control DNA from CPCC 204095/FKO/pSET152 (lanes 1–3). Total DNA prior to immunoprecipitation (lanes 1 and 4) was used as positive control for PCR. DNA treated with anti-Flag antibody (lanes 2 and 5) or with IgG antibody (lanes 3 and 6) was analyzed by PCR. (**D**) IsaF consensus binding sites revealed by MEME analysis. The data for this logo consist of four IsaF binding sites including the promoter regions of *isaD*, *isaH*, *isaL*, and *isaS*. The height of each letter is proportional to the frequency of the base. The grey shades and arrows indicate forward repeats. (**E**) Electrophoretic mobility shift assays (EMSAs) were used to analyze the interaction of the promoter regions with purified His-SUMO-tagged IsaF. The amounts of IsaF in the reaction are indicated above each lane. The “competitor” lane contains 10 µl IsaF with a 200-fold excess of unlabeled specific competitor

To further investigate the regulatory function of the IsaF protein in the biosynthesis of isatropolone, we examined the involvement of the *isaF* gene in transcription regulation of *isa* cluster. Gene expression analysis was conducted using reverse transcription-quantitative PCR (RT-qPCR). In the *isaF* overexpression strain, the expression levels of the type II PKS core genes *isaG*, *isaH*, *isaI*, *isaL*, and the oxygenase gene *isaP* in the *isa* gene cluster, which are responsible for the assembly of the core skeleton of tropolone, were upregulated by 2–3 times due to the *isaF* overexpression (Fig. 3A). Additionally, the mRNA levels of most *isa* genes, such as *isaG*, *isaH*, *isaI*, *isaL*, *isaP*, *isaS*, and *isaT*, decreased in FKO and were upregulated in the *isaF* complementary strain as the *isaF* reintroduced into FKO (Fig. 3B). The above results indicate that IsaF is a typical positive pathway-specific regulator, which regulates isatropolone biosynthesis by activating the transcription of the biosynthetic structural genes.

To determine which biosynthetic genes in the *isa* cluster are directly activated by IsaF, the DNA-binding activity of IsaF in vivo was confirmed using a chromatin immunoprecipitation PCR (ChIP-PCR) assay in the genetically modified strain CPCC 204095/FKO. A pSET152-derivative plasmid encoding IsaF tagged with 3×Flag epitope at its C-terminus was introduced into the FKO strain. The western blot and HPLC analysis showed that IsaF tagged with 3×Flag was effectively expressed and maintained the original function of IsaF within the FKO strain (Additional file 1: Fig. S6A, S6B). The binding activity of IsaF was determined by the amplification of the promoter regions of interest genes that could be immunoprecipitated by anti-Flag antibody. Subsequently, it was found that the promoter regions of *isaD-E*, *isaH*, *isaL* and *isaS-T* were precipitated by the 3×Flag tagged IsaF (Fig. 3C) while other detected promoter regions not (Additional file 1: Fig. S6C). Furthermore, the alignment of the four known IsaF binding sequences (*isaD-E*p, *isaH*p, *isaL*p and *isaS-T*p) revealed a consensus sequence of 5’-TCGAASN_6_TCDVCV-3’, characterized by a 6 bp forward repeat sequence separated by a 6 bp linker (Fig. 3D), which also represents a typical SARP family consensus sequence.

Then a series of electrophoretic mobility shift assays (EMSAs) was performed to verify whether IsaF can directly bind to the promoters of biosynthetic genes in vitro. IsaF was expressed in *E. coli* BL21 (DE3) as His-SUMO-tagged protein with a predicted molecular mass of 47 kDa and was purified by nickel affinity chromatography (Additional file 1: Fig. S7A). DNA fragments of intergenic regions containing possible promoters of interest genes were generated as probes of *isaD-E*p, *isaF*p, *isaG*p, *isaH*p, *isaJ*p, *isaL*p, *isaP*p, *isaQ*p, *isaR*p, *isaS-T*p, *isaU-V*p, and *isaW*p (Additional file 1: Fig. S7B). EMSA results showed that the purified His-SUMO-tagged IsaF can bind to *isaD-E*p, *isaH*p, *isaL*p and *isaS-T*p to form a complex(es) (Fig. 3E), but not to the other 8 probes (Additional file 1: Fig. S7C). The bindings were enhanced by increasing the amount of IsaF, and the shifting of the probes decreased when the unlabeled specific competitor DNAs were added in excess to the binding reactions, suggesting the bindings were specific.

The specific bindings of IsaF to several promoter regions of the *isa* cluster in vitro and in vivo, along with the results of overexpression and disruption of *isaF*, indicated that IsaF positively regulates isatropolone biosynthesis by directly binding to the promoter regions of these biosynthetic genes. As biosynthetic genes are organized in operons in the biosynthetic gene cluster, binding of IsaF to the promoters of *isaD-E*, *isaH*, *isaL*, and *isaS-T* may activate multiple transcription units from *isaD* to *isaT* for the production of isatropolones. Consistent with this, the results of *isaC* knockout showed that IsaC is not required for the biosynthesis of isatropolones (Additional file 1: Fig. S8).

### IsaJ regulates production of isatropolone C

**Fig. 4:**
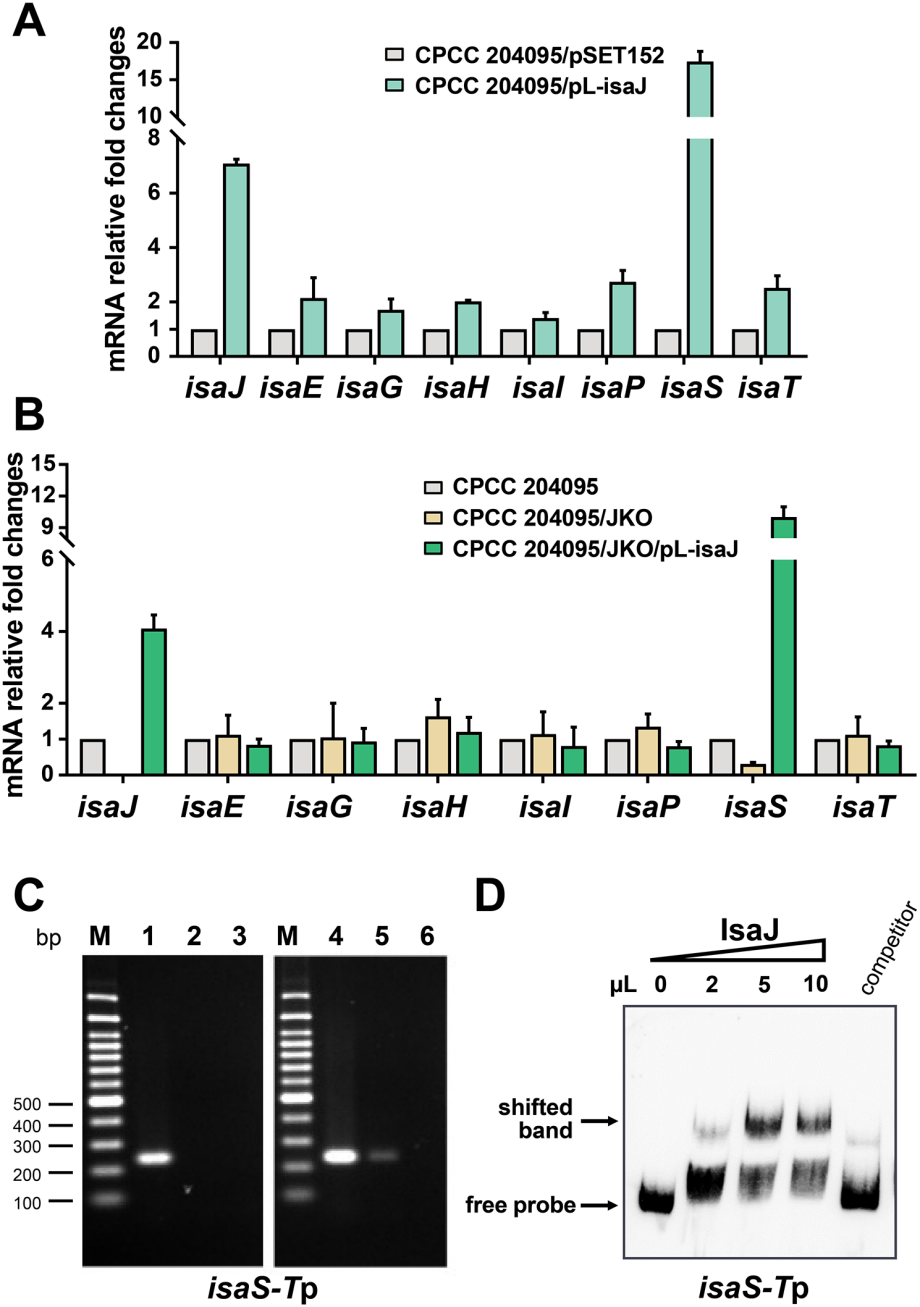
Identification of the target genes regulated by IsaJ. (**A**, **B**) Transcriptional analysis of *isa* genes in the *isaJ* overexpression strain (CPCC 204095/pL-isaJ), and *isaJ* gene knockout strain (JKO) and genetic complementary strain (JKO/pL-isaJ) by RT-qPCR analysis. The relative mRNA level of the target genes was normalized to the principal sigma factor gene *hrdB*. The relative expressional level of each sample was represented as the value related to the control strain CPCC 204095/pSET152 or CPCC 204095. Values are presented as mean ± SEM (two biological repeats for each strain). (**C**) ChIP-PCR analysis of Flag-tagged IsaJ. PCR using primers flanking target promoter regions were performed with immunoprecipitated DNA from CPCC 204095/JKO/pL-isaJ-Flag (lanes 4–6) and negative control DNA from CPCC 204095/JKO/pSET152 (lanes 1–3). Total DNA prior to immunoprecipitation (lanes 1 and 4) was used as positive control for PCR. DNA treated with anti-Flag antibody (lanes 2 and 5) or with IgG antibody (lanes 3 and 6) was analyzed by PCR. (**D**) EMSA to analyze the interaction of the promoter regions with purified IsaJ. The amounts of IsaJ in the reaction is indicated above the lanes. The “competitor” lane contains 10 µl of IsaJ with a 200-fold excess unlabeled specific competitor

IsaJ has been predicted to be another SARP family regulator with an N-terminal of OmpR-like DNA binding domain in the *isa* cluster and conserved in the gene clusters of *ist*, *rub*, and *rbl*. As shown in Fig. 2E and F, the overexpression of *isaJ* almost abolished the production of isatropolone A, while conversely, the disruption of *isaJ* significantly decreased isatropolone C production and led to an accumulation of isatropolone A. To further investigate the regulatory mechanism of *isaJ* gene on the production of isatropolone A and C, we subsequently investigated the changes in transcriptional levels of the *isa* genes in *isaJ* overexpression and knockout strains. Notably, transcriptional analysis showed that the overexpression of *isaJ* could significantly increase the transcriptional level of *isaS*, a predicted cytochrome P450 monooxygenase gene, while the deletion of *isaJ* led to the inhibition of the transcription of *isaS* (Fig. 4A and B). The transcript analysis showed that IsaJ did not affect the transcription of any other *isa* genes detected, which are responsible for catalyzing the formation of the aglycone of isatropolones (Fig. 4A and B).

Furthermore, ChIP-PCR and EMSAs analyses were performed to explore whether IsaJ can activate the transcription of *isaS* by directly binding to its promoter region. Consistent with RT-qPCR results, IsaJ was found to bind to the fragment of *isaS-T*p in vivo and in vitro (Fig. 4C and D), but to not any other *isa* genes detected (Additional file 1: Fig. S9 and S10). This is a rare example where regulation of secondary metabolism from a cluster-situated transcriptional regulator is limited to only one specific gene encoding a biosynthetic enzyme. In conclusion, IsaJ affects the production of isatropolone C by regulating the transcription of the *isaS* gene.

As isatropolone A and C differ in structure by only one hydroxyl group, an oxygenase might be responsible for the conversion. In the *isa* cluster, there are three oxygenase genes, *isaE*, *isaP*, and *isaS*. IsaP might be involved in the biosynthesis of the aglycone moiety of isatropolones as suggested by the heterologous expression of *istG-R* from *Streptomyces* Gö66 in *S. lividans* [8]. The results of the regulatory target gene of IsaJ unexpectedly provide a clear clue that IsaS, but not IsaE, is responsible for the C-3 hydroxylation of isatropolone C.

### IsaS is responsible for C-3 hydroxylation of isatropolone A to C

The gene *isaS*, located in the interior of the *isa* cluster, encodes a cytochrome P450 monooxygenase consisting of 409 amino acids. Cytochromes P450 proteins are a family of heme-containing oxygenase, known for their ability to catalyze various substances, including secondary metabolites, through reactions such as *O*-dealkylation, *S*-dealkylation, epoxidation, and mostly hydroxylation [20]. Through BLAST analysis, it was found to have a 39% amino acid identity and 53% similarity to the cytochrome P450 enzyme CYP158A1, which can catalyze the flaviolin dimerization reactions in *S. coelicolor* [21]. IsaS also displays end-to-end 31% sequence identity and 45% similarity to the product of *pimD* [22], which encodes the cytochrome P450 epoxidase converting de-epoxypimaricin into pimaricin in *S. natalensis* (Additional file 1: Fig. S11). The 3D structure of IsaS predicted by Swiss Model has typical P450 structural fold characteristics (Additional file 1: Fig. S11C).

**Fig. 5:**
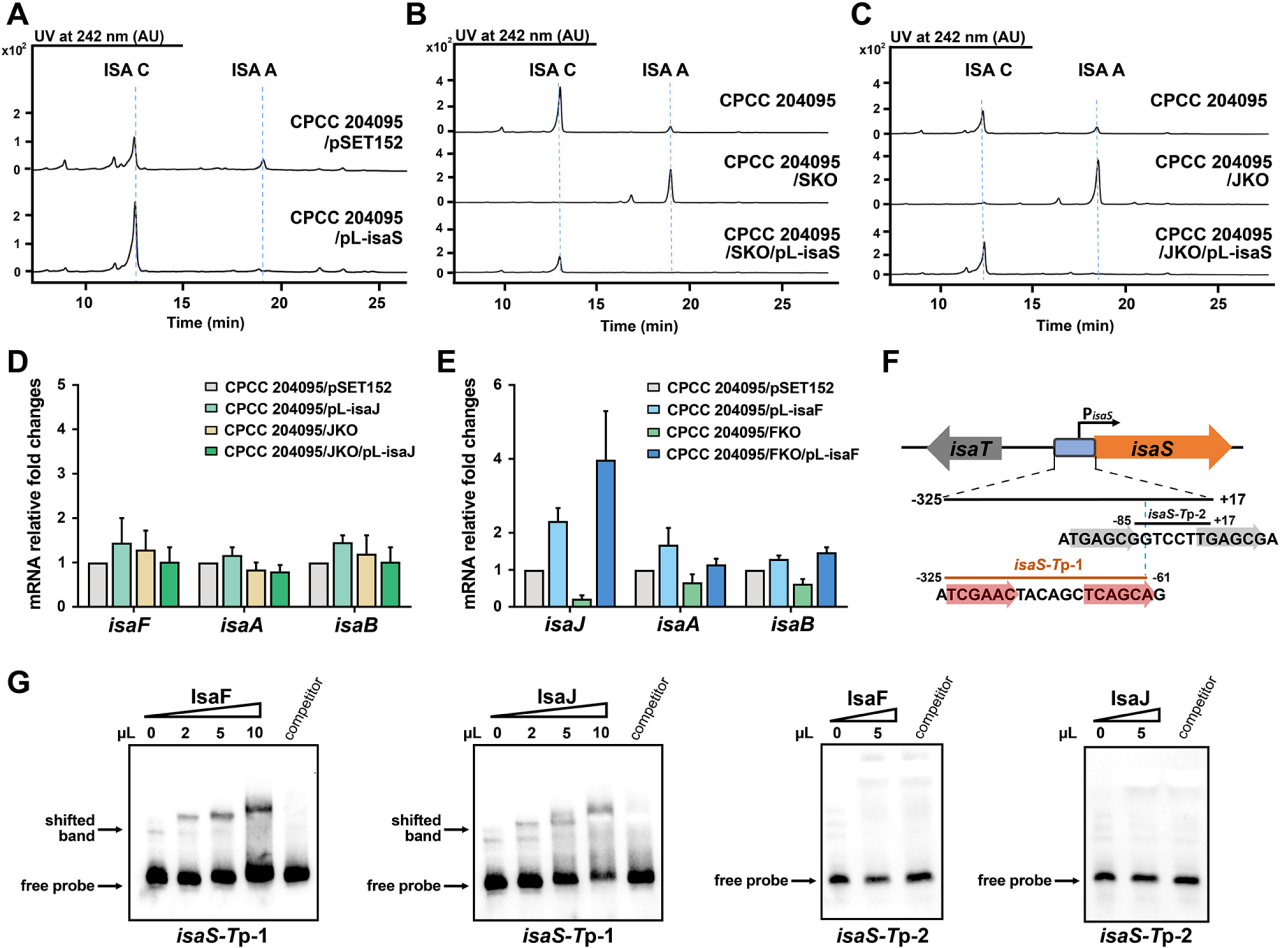
A regulatory cascade of IsaF/J/S for the biosynthesis of isatropolone. (**A**, **B**, **C**) HPLC analysis was used to monitor the production of isatropolone A (ISA A) and isatropolone C (ISA C) in the wild type strain (CPCC 204095), the empty vector control strain (CPCC 204095/pSET152), the overexpression strain (CPCC 204095/pL-isaS), gene knockout strains (SKO and JKO), and genetic complementary strains (SKO/pL-isaS, JKO/pL-isaS). (**D**, **E**) Transcriptional analysis of *isaF*, *isaJ*, *isaA* and *isaB* in the overexpression strains (CPCC 204095/pL-isaJ, CPCC 204,095/pL-isaF), gene knockout strain (JKO, FKO), and genetic complementary strains (JKO/pL-isaJ, FKO/pL-isaF) was performed using RT-qPCR analysis. The relative mRNA level of the target genes was normalized to the principal sigma factor gene *hrdB*. The relative expressional level of each sample was represented as the value related to the control strain CPCC 204095/pSET152. Values are presented as mean ± SEM (two biological repeats for each strain). (**F**) Schematic representation for two overlapped probe fragments in EMSA. Arrows indicate possible forward repetitive binding sequences. (**G**) EMSA was used to analyze the interaction of the fragmented promoter regions with purified IsaF or IsaJ. The amounts of IsaF or IsaJ in the reaction were indicated above the lanes. For *isaS*-*T*p-1, the “competitor” lane contains 10 µl of IsaF or IsaJ with a 200-fold excess of unlabeled specific competitor. For *isaS*-*T*p-2, the “competitor” lane contains 5 µl of IsaF or IsaJ with a 200-fold excess of unlabeled specific competitor

To confirm the function of IsaS as a cytochrome P450 monooxygenase responsible for the C-3 hydroxylation of isatropolone C in vivo, overexpression and knockout strains were constructed (Additional file 1: Fig. S12) and the fermentation products were analyzed. The *isaS* overexpression strain showed an increase in isatropolone C production, while the knockout strain resulted in a higher level of isatropolone A accumulation but no isatropolone C production further (Fig. 5A and B). Reintroducing *isaS* into the knockout strain led mainly the production of isatropolone C (Fig. 5B). These results confirmed that IsaS is responsible for the formation of C-3 hydroxyl of isatropolone C.

The enzymatic function of IsaS prompts us to consider why IsaJ evolved to specifically regulate the transcription of this particular gene in the isatropolone natural producing strain. Sarwar et al. reported that isatropolone C possessed anti-*Streptomyces scabies* activity [23]. Therefore, we assessed the anti-*S. scabies* activity of isatropolone A and C, and the results indicated that the activity of isatropolone C is considerably higher than that of isatropolone A (Additional file 1: Fig. S13). This implies that for the producing strain, isatropolone C might be produced to compete with other streptomycetes in the natural environment. The direct regulation of IsaS by IsaJ may ensure isatropolone A to be transformed into the more effective anti-*Streptomyces* compound only when necessary, thus minimizing its self-toxicity.

### A cascade of IsaF/J/S for regulating the biosynthesis of isatropolone

The two SARP transcriptional regulatory factors, IsaF and IsaJ, are involved in the biosynthesis of isatropolone in apparently different regulatory modes. However, the hierarchy of these two regulators in the pathway-specific regulatory cascade for the biosynthesis of isatropolone remains to be determined. Thus, we extended our analysis of the transcript levels of regulatory genes in *isaF* and *isaJ* knockout strains. The transcription level of *isaF* did not change in the JKO strain compared to the wild type, suggesting *isaF* was not the target gene of IsaJ (Fig. 5D). On the other hand, the transcription level of *isaJ* was significantly lower in FKO strains and approximately 2-fold upregulated in the overexpression strains of *isaF* (Fig. 5E). These results suggested that IsaF acts positively on IsaJ and occupies a higher rung than IsaJ does in the hierarchy of *isa* regulatory cascade.

From *isaS* perspective, decreased transcripts were observed in both *isaF* and *isaJ* knockout strains, whereas increased transcripts were detected in the overexpression strains (Figs. 3A and B and 4A, and 4B). These results indicated that *isaS* is the target gene of *isaF* and *isaJ* in isatropolone biosynthesis. ChIP-PCR and EMSA results showed that both IsaF and IsaJ can bind to the promoter region of the *isaS* gene in vitro and in vivo (Figs. 3C and E and 4C, and 4D). Another confirmation that *isaS* was directly controlled by IsaJ came from the *in trans* complementation of IsaJ knockout mutant with *isaS* gene. Isatropolone C was produced again after the introduction of constitutively overexpressed *isaS* into JKO (Fig. 5C).

Two forward repeat sequences in *isaS-T*p have been predicted to be highly similar to the binding consensus sequence of the SARP family proteins (Fig. 5F). To investigate whether IsaF and IsaJ regulate the transcription of *isaS* by binding to different regions of *isaS-T*p, we performed a series of EMSAs using two overlapping fragments that were separated from the long divergent intergenic region between *isaS* and *isaT*, each containing one of the forward repeat sequences. Our findings revealed that a DNA fragment (*isaS-T*p-1) extending from position − 325 to -61 (where + 1 represents the first A of translation start codon ATG of *isaS*), with a TCGAACN_6_TCAGCA forward repetitive sequence, was bound by both IsaF and IsaJ (Fig. 5G). This suggests that IsaJ and IsaF both bind to the same promoter region to jointly regulate the expression of the *isaS* gene.

Most well-studied pathway-specific regulators, such as ActII-ORF4, CdaR, RedD and AfsR, are members of the SARP family and serve as pathway-specific or global regulators in the biosynthesis of specific antibiotics [14]. In cases where a gene cluster contains multiple SARPs, it is possible that one of them may not be essential for the biosynthesis of secondary metabolites, or there may be a hierarchical regulatory cascade among them controlling the production of metabolites, usually involving the yield of secondary metabolites without affecting their chemical structure [24]. It is uncommon that one of the SARP regulators serves as a master regulator activating metabolite production, while another determines the conversion of different products by regulating transcription of a single structure gene, as observed in the case of IsaF and IsaJ. Additionally, there is a cascade regulatory relationship between these two regulators, with IsaF acting as a master switch and IsaJ as a lower-level regulator for isatropolone C production. Although both IsaF and IsaJ can bind to the promoter region of the *isaS* gene and activate its transcription, the absence of IsaJ sharply reduces the initiation of *isaS* transcription, even in the presence of IsaF. Further research is required to fully elucidate the collaborative mechanism of IsaF and IsaJ in finely regulating the expression of *isaS*. This novel regulatory mechanism of two SARPs in isatropolone biosynthesis will serve as a guide for further rational engineering of *Streptomyces* strains to achieve high-level and selective production of various products.

### Rational engineering of *Streptomyces* strains for high-level and selective isatropolone A production

**Fig. 6:**
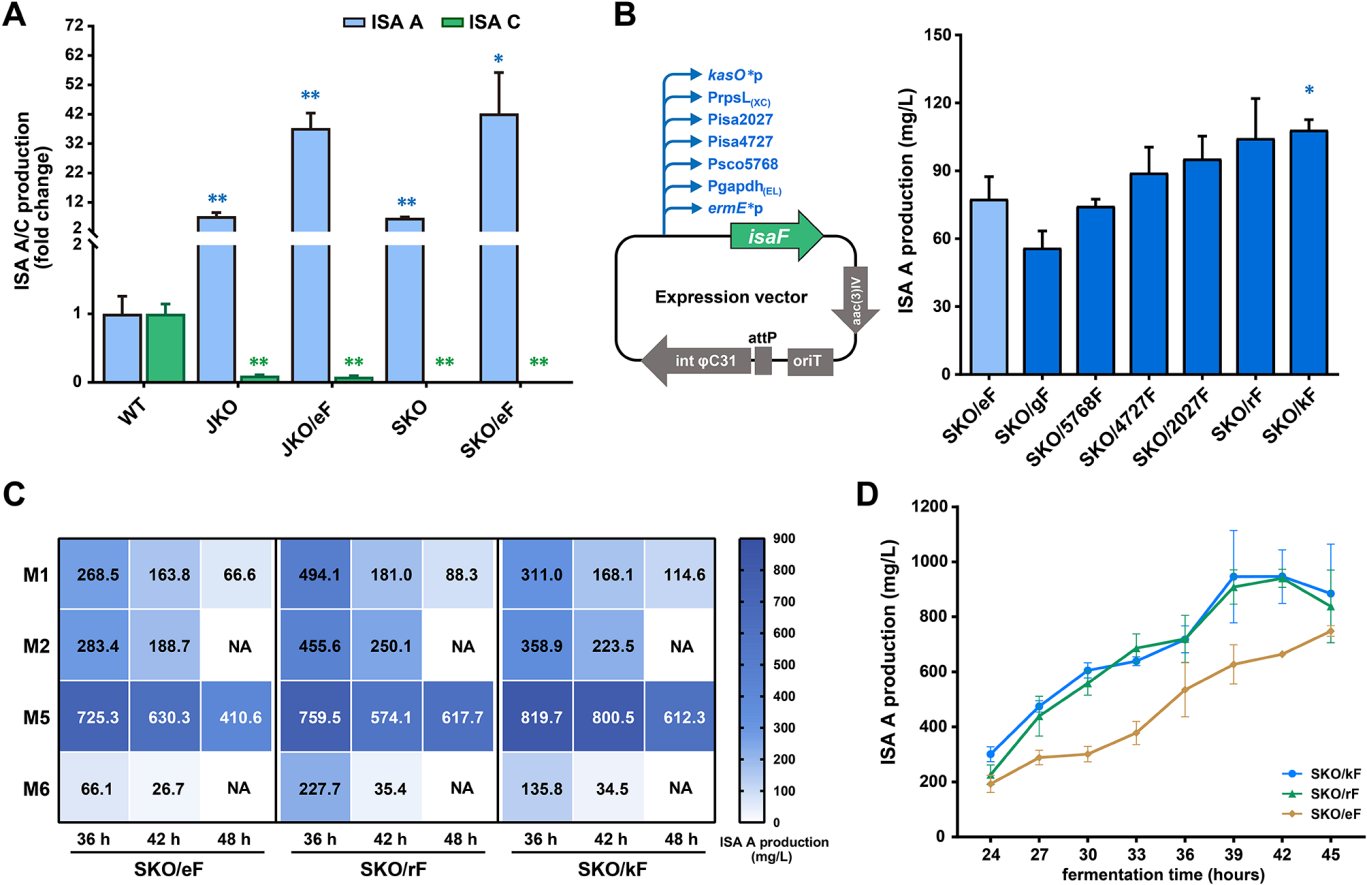
Rational engineering of *Streptomyces* strains for high-level and selective isatropolone A production. (**A**) Quantification the production of isatropolone A (ISA A) and isatropolone C (ISA C) in the wild type strain (CPCC 204095) and its derivatives was performed using HPLC-MS analysis. The relative yields of ISA A and C in each strain were compared with those in the wild type strain (Student’s *t* test, *p < 0.05, **p < 0.01). Values are presented as mean ± SEM (three independent conjugants for each strain). (**B**) Schematic representation of the construction of SKO derivatives with different promoters driving the expression of *isaF*. The production level of ISA A in these derivatives was determined by HPLC-MS analysis. Values are presented as mean ± SEM (three independent conjugants for each strain). The yield of ISA A in each strain was compared with those in the SKO/eF strain (Student’s *t* test, *p < 0.05). (**C**) Heat maps showing the titers of isatropolone A in three SKO derivatives, each with different promoters driving the expression of *isaF*, across various combinations of different media and fermentation time points. “NA” indicates that ISA A was not detected. (**D**) The production curves of isatropolone A were obtained from three recombinant strains on M5 agar medium. Values are presented as mean ± SEM (two independent conjugants for each strain)

Given our understanding of the regulatory mechanism of isatropolones biosynthesis, we aimed to engineer strains for the targeted high-level production of isatropolone A. In the naturally producing strain *Streptomyces* sp. CPCC 204095, the yields of isatropolones and the proportion of isatropolone A (usually as a minor component) vary with a different number of seeding spores, fermentation time and batches on ISP2 agar plates. The above work showed there was a significant increase in the production of isatropolone A in the JKO and SKO strains, being 7.3 times and 6.8 times higher than that in the wild type strain, respectively (Fig. 6A). To further enhance the yield of isatropolone A, we introduced an additional overexpression plasmid (pSET-eF) containing the positive regulatory gene *isaF* under ermE*p into the JKO and SKO strains, resulting in the construction of the JKO/eF and SKO/eF strains, respectively. Under the same fermentation conditions, both strains exhibited significantly higher production of isatropolone A, approximately 5.1 times and 6.3 times greater than the JKO and SKO strains, and approximately 40 times higher than in *Streptomyces* sp. CPCC 204095 (Fig. 6A). Notably, in the SKO strains, the production of isatropolone C was completely abolished (Fig. 6A), indicating that SKO/eF, with production of isatropolone A reaching 77.5 mg/L, might be more suitable as a host for further improvement of isatropolone A production.

Subsequently, to pursuing further enhancement of the expression level of the *isaF* gene in SKO strains, six different promoters were used to substitute for ermE*p in the overexpression plasmid pSET-eF. The six promoters included four well-known constitutive promoters, namely, Pgapdh_(EL)_ [25], PrpsL_(XC)_ [25], kasO*p [26], and Psco5768 [27], as well as the promoters of the RNA polymerase sigma factors in CPCC 204095, Pisa2027, and Pisa4727 (Fig. 6B, Additional file 1: Fig. S14). Under the same fermentation conditions, PrpsL_(XC)_ and kasO*p showed superior performance compared to ermE*p in pSET-eF, and the modified strains SKO/rF and SKO/kF exhibited an increase in isatropolone A production by 1.34 and 1.39 times, respectively (Fig. 6B). Consequently, we selected these two strains with higher isatropolone A production for further investigation of the optimal fermentation conditions.

After engineering the high-yield strains SKO/eF, SKO/rF and SKO/kF, we conducted a comparison of various culture media formulations and their effects on isatropolone A titers. To achieve a more stable and higher yield, we expanded the fermentation system from 9 cm to 15 cm diameter plates, increased spore inoculation to 10^9^ spores/plate, and investigated 6 different media and 3 fermentation time points. The selection of culture media was based on those used by different bacteria in the literature, mainly for the production of tropolones, including M1 (i.e., ISP2), M2 [8], M3 [9], M4 [28], M5 [13], and M6 [29], each containing different carbon and nitrogen sources. Isatropolone A production was found to be extremely low in M3 and M4 media after 48 h of fermentation and the data was not shown. The results (Fig. 6C) showed that the three engineered strains cultured in M5 media exhibited significantly higher yields compared to those in other media, with the optimal fermentation time being 36 h in most cases. Additionally, it was observed that with an extension of fermentation time, isatropolone A production decreased, potentially due to the non-enzymatic transformation of isatropolones with amine-containing compounds to corresponding isarubrolones [8]. Additional fermentation batch with two independent conjugants for each recombinant construct confirmed the reliable high yield in M5 medium (Fig. 6D). With the inoculum spore concentration of 10^9^ spores/plate, the highest production of isatropolone A was achieved in the strain SKO/kF, reaching an average of 946.3 mg/L. It is interesting that increasing the spore inoculation of the SKO/kF to 10^10^ spores/plate accelerated the peak production of isatropolone A by 6 h, with the overall yield remaining largely similar (980.8 mg/L) (Additional file 1: Fig. S15). This is currently the highest reported production of isatropolones, much higher than a previous report which utilized a 10 L fermenter [8]. We provide a genetic strategy based on regulatory mechanisms to engineer *Streptomyces* sp. CPCC 204095 for the targeted high-level production of isatropolone A, which could potentially be applied to achieve high-yield production of other specific natural products in the future.

## Conclusion

In this study, we have firstly deciphered the biosynthetic regulation mechanism of isatropolones. Within the regulatory system, the SARP family regulator, IsaF, is identified as a pathway-specific master regulator responsible for activating the biosynthetic gene cluster. Another SARP regulator, IsaJ, specifically controls the transcription of P450 monooxygenase IsaS, which is responsible for the C-3 hydroxylation of isatropolone C. Based on this acquired knowledge of the biosynthetic mechanisms, we successfully engineered the high-producing strain SKO/eF, which exclusively produces the anti-leishmania compound isatropolone A. Additional efforts were made to optimize the promoters for driving the expression of *isaF* in SKO, resulting in the creation of two recombinant strains (SKO/rF and SKO/kF) with higher isatropolone A production. Ultimately, through the use of culture media from reported tropolone-producing cultures, we achieved targeted high-yield production of isatropolone A at 980.8 mg/L, the highest reported titer to date. This achievement paves the way for future drug development, providing sufficient quantities of isatropolone A for pharmacological, pharmacokinetic, and toxicological research.

## Materials and methods

### Strains, plasmids, and growth conditions

*Streptomyces* sp. CPCC 204095 and its derivatives were grown on tryptic soy broth (TSB, BD, USA) and ISP2 agar (yeast extract 4 g/L, malt extract 10 g/L, glucose 4 g/L, agar 20 g/L) for routine cultivation, on mannitol soya flour (MS) agar [30] for sporulation and conjugation, in TSB liquid medium for the isolation of genomic DNA, and on ISP2 agar for RNA extraction. *Escherichia coli* ET12567/pUZ8002 [30] was used for conjugal transfer DNA into *Streptomyces*. *E*. *coli* BL21 (DE3) (Novagen, Madison, USA) was used as the host strain to express IsaF/J proteins. They were grown at 37 ^o^C in Luria-Bertani medium (LB). Strains, plasmids, and primers used in this study are listed in Additional file 1: Table S1 and S2.

### Gene inactivation, complementation and overexpression

Gene disruption was carried out by the in-frame deletion method. To construct the *isaF* knockout strain (FKO), two 1.3-kb fragments flanking *isaF* were amplified by PCR using two primer pairs (isaFL_F/ isaFL_R and isaFR_F/isaFR_R) (Additional file 1: Table S2). These fragments were digested by *Hin*dIII/*Nde*I or *Nde*I/*Xba*I, and then ligated into the corresponding sites of pKC1139, yielding the plasmid pKC-isaF with an internal 708-bp deletion of *isaF*. The pKC-isaF was transformed into *E. coli* ET12567/pUZ8002, and then conjugated into *Streptomyces* sp. CPCC 204,095. The resulting mutant FKO was further confirmed by PCR verification. The strategy for constructing *isaJ* knockout strain (JKO) was consistent with the approach used to obtain FKO.

To construct *isaS* knockout strain (SKO), the 1.5-kb fragment and the 1.2-kb fragments flanking the *isaS* gene were amplified by PCR with specific primer pairs (isaSL_F/isaSL_R and isaSR_F/isaSR_R) (Additional file 1: Table S2). These fragments were then digested by *Hin*dIII/*Nde*I and *Nde*I/*Xba*I, and a 0.9-kb DNA fragment containing the thiostrepton resistant gene coding region was inserted between them. The resulting construct was then ligated into pKC1139, which had been digested by *Hin*dIII/*Xba*I, to obtain pKC-isaS. Following the transformation into *E. coli* ET12567/pUZ8002, disruption plasmids were conjugated into *Streptomyces* sp. CPCC 204095. The double crossover strains were selected as thiostrepton-resistant but apramycin-sensitive mutants, which were confirmed by PCR amplification. The strategy for constructing *isaA* knockout strain (AKO), *isaB* knockout strain (BKO), and *isaC* knockout strain (CKO) was consistent with the approach used to obtain SKO.

For the overexpression and complementation of *isaF*, a 903-bp DNA fragment containing the complete *isaF* coding region was amplified using isaF_F and isaF_R as primers (Additional file 1: Table S2) and then cloned into the *Nde*I and *Bam*HI sites of pICLSet [31] under the control of a strong constitutive promoter ermE*p by Gibson assembly. The resulting plasmid pL-isaF was introduced into FKO by conjugal transfer, generating the complementary strain CPCC 204095/FKO/pL-isaF. Plasmid pL-isaF was introduced into the wild type strain CPCC 204095 to obtain the overexpressed strain CPCC 204095/pL-isaF. Following the mentioned procedures above, we constructed complementation or overexpression strains for *isaA*, *isaB*, *isaJ* and *isaS*.

### Analysis of isatropolone production

*Streptomyces* sp. CPCC 204095 and its derivatives were grown on ISP2 agar plates for isatropolone A/C production. 50 µl spore suspension (∼ 2 × 10^8^ cfu/ml) was spread on a diameter of 9 cm dish containing 12 ml fermentation medium. All fermentation cultures were incubated at 28 °C for 24, 36–48 h. After fermentation, 24 ml of ethyl acetate was added to extract products at room temperature in dark for 24 h. Afterwards, 1 ml extracted sample was evaporated and redissolved in 100 µl methanol, filtered using a 0.22 μm microporous membrane, and analyzed by HPLC-MS (Agilent 1100 series HPLC coupled to Agilent 6410 Triple Quadrupole mass spectrometer). The HPLC conditions were as follows: Agilent Eclipse Plus C18 column (150 mm × 4.6 mm, 5 μm), mobile phase solvent A was 100% acetonitrile, solvent B was water with 0.1% acetic acid. The HPLC program included column elution with a linear gradient of 15 ∼ 70% of solvent A over 30 min at 25 °C. The flow rate was set at 1 ml/min. The products were monitored at 242 nm. MS spectra data were collected in the positive-ion mode. The isatropolone C and isatropolone A peaks were detected from extracted ion chromatogram (EIC) at *m/z* 473 [M + H]^+^ and 457 [M + H]^+^, respectively. For quantitation of isatropolone C and isatropolone A, linear calibration curves were obtained by plotting the standard concentration versus the corresponding LC peak area ratios. The standards were purified from fermentation broth according to the methods reported previously [10].

### Transcriptional analysis by reverse transcription-quantitative PCR (RT-qPCR)

Total RNA was isolated from *Streptomyces* sp. CPCC 204095 and its derivatives after 36 h of growth in fermentation medium with cellophane using TRIzol reagent (Invitrogen, USA) and chloroform followed by a PureLink^™^ RNA Mini Kit (Invitrogen, USA). The first-strand synthesis of cDNA was achieved from 1 µg of each RNA sample using TransScript® One-Step gDNA Removal and cDNA Synthesis SuperMix (TransGen, China). qPCR was performed on the CFX96 Touch Real-Time PCR Detection System (Bio-Rad). Each reaction volume of 25 µl was composed of 2.5 µl cDNA, 12.5 µl Fast Start Universal SYBR Green Master ROX (Roche, Switzerland), 0.25 µM each of forward and reverse primers and 5 µl RNase-free water. qPCR conditions were as follows: 94 °C for 10 min, 40 cycles of 94 °C for 30 s, 60 °C for 30 s and 72 °C for 30 s. The *hrdB* gene encoding the major sigma factor of *Streptomyces* was used as the internal control according to Pfaffl’s method [32].

### Expression and purification of IsaF and IsaJ

The *isaF* and *isaJ* coding sequences were amplified from *Streptomyces* sp. CPCC 204095 genomic DNA with primers (Additional file 1: Table S2) and then cloned into the *Hin*dIII and *Bam*HI sites of pET28a-SUMO by Gibson assembly. Then these plasmids were introduced into *E. coli* BL21(DE3) to express IsaF and IsaJ as fusion proteins with the N-terminal His-SUMO-tag. The transformed strains were cultured in LBBS liquid medium at 37 °C until OD_600_ of 0.4 ∼ 0.6, and then induced with isopropyl-β-D-thiogalactopyranoside (IPTG) at a final concentration of 0.5 mM at 16 °C for 72 h. The cells were harvested by centrifugation (14,000 rpm, 30 min, 4 °C), and resuspended in binding buffer (20 mM Na_3_PO_4_, 500 mM NaCl, pH 7.4), then lysed by homogeniser (Fastprep, 4.0 m/sec, 20s). The lysate was centrifuged (12,000 rpm, 30 min, 4 °C), and the His-SUMO-tagged proteins present in the supernatant were purified using HisTrap^TM^  FF crude kit (GE Healthcare), eluted with the elution buffer (20 mM Na_3_PO_4_, 500 mM NaCl, and 500 mM imidazole, pH 7.4), and desalted by PD-10 Desalting Columns (GE Healthcare) against 1× TGEK buffer (250 mM Tris, 50% Glycerol, 5 mM EDTA, 500 mM KCl, pH 8.0). The concentration of purified proteins was quantified by BCA assays, and the purity was assessed by SDS-PAGE analysis.

### Chromatin immunoprecipitation (ChIP) -PCR analysis

To construct the complementary strains expressing IsaF-3×FLAG and IsaJ-3×FLAG, the *isaF* and *isaJ* coding sequences were amplified with primers (Additional file 1: Table S2) fused with 3×Flag tag and inserted into the pICLSet plasmid using Gibson assembly. The resulting plasmids, pL-isaF-Flag and pL-isaJ-Flag, were introduced into the FKO and JKO strain through conjugation, resulting in the complementary strains CPCC 204095/FKO/pL-isaF-Flag and CPCC 204095/JKO/pL-isaJ-Flag.

To prepare samples for immunoprecipitation, 1 × 10^8^ spores of the CPCC 204095/FKO/pL-isaF-Flag and CPCC 204095/JKO/pL-isaJ-Flag strains were grown on ISP2 agar covered with cellophane for 36 h. The harvested mycelium was treated with 1% (vol/vol) formaldehyde for 30 min and gently shaken to cross-link the protein with DNA, followed by glycine treatment and washing twice with cold phosphate-buffered saline (PBS) solution. The treated mycelium was stored at -80 °C before being resuspended in Lysis buffer (50 mM Tris, 150 mM NaCl, 1 mM EDTA, 1% Triton X-100, 1× Protease Inhibitor mix, pH 7.4) and then sonicated to fragment DNA ranging from 100 to 500 bp on average. The resulting cellular extract supernatant was used to perform immunoprecipitation by incubating with anti-flag M2 affinity antibody beads or anti-IgG affinity antibody beads for overnight at 4 °C. Beads was washed five times with lysis buffer containing 0.5 M NaCl free of protease inhibitor, followed by elution with Elution buffer (1% SDS, 100 mM NaHCO_3_, 10 mM EDTA, pH 8.0). The cross-links were reversed by incubating at 65 °C overnight with 5 µl of 5 M NaCl. The ChIP-DNA was then purified and used for subsequent PCR analysis after protein removal using Proteinase K digestion followed by phenol-chloroform extraction.

### Electrophoretic mobility shift assays (EMSAs)

DNA fragments of the promoters were obtained by PCR using the primers listed in Additional file 1: Table S2 and were labeled with biotin at their 5’-end as probes in the EMSAs. The binding reaction system had a total volume of 20 µL and consisted of 20 fmol of labeled probe, 50 to 500 nm of purified protein, 2 µL of 10 × binding buffer (100 mM Tris-HCl, 500 mM KCl, 10 mM DTT, pH 7.5). As controls, unlabeled specific probes (> 200 fold of the labeled probes) were added. After a 30 min incubation at room temperature, the samples were migrated to a native 5% polyacrylamide gel (buffered with 0.5 × TBE and run at 4 °C, 120 V) and then transferred to a nylon membrane (Amersham Biosciences) by electrophoretic transfer. The shift of biotin end-labeled probes was detected using the Lightshift chemiluminescent EMSA kit (Thermo Scientific) following the manufacturer’s instructions.

### Construction of the engineered strains for high-level production of isatropolone A

The coding region of *isaF* was PCR-amplified from *Streptomyces* sp. CPCC 204095 using the primers isaF_F2 and isaF_R2 and then cut with *Spe*I and *Not*I. The promoter ermE*p was PCR-amplified using the primers ermE*p_F and ermE*p_R and cut with *Xba*I and *Spe*I. The promoter and the *isaF* coding region were ligated together into the *Xba*I and *Not*I sites of pSET152 in a three-piece ligation reaction to generate pSET-eF. The pSET-eF was transformed into *E. coli* ET12567/pUZ8002 and was conjugated into *Streptomyces* sp. CPCC 204095/JKO and SKO to generate JKO/eF and SKO/eF, respectively.

For construction of SKO derivatives with different promoters driving the expression of *isaF*, six promoters (Pgapdh_(EL)_ [25], PrpsL_(XC)_ [25], kasO*p [26], Psco5768 [27], Pisa2027 and Pisa4727) were obtained by PCR amplification with primers listed in Additional file 1: Table S2. These promoters were cut with *Xba*I and *Spe*I and ligated together into pSET-eF to replace the promoter of ermE*p to generate pSET-gF, pSET-rF, pSET-kF, pSET-5768 F, pSET-2027 F and pSET-4727 F. All derived plasmids were confirmed by PCR and restriction enzyme digestion analysis. Then these plasmids were transformed into *E. coli* ET12567/pUZ8002 and conjugated with *Streptomyces* sp. CPCC 204095/SKO to generate SKO/gF, SKO/rF, SKO/kF, SKO/5768F, SKO/2027F, and SKO/4727F.

Six fermentation media were screened for isatropolone A production. Compositions of these six culture media were as follows: Medium M1 (ISP2), M2 [8] (yeast extract 4 g/L, malt extract 10 g/L, glucose 4 g/L, D-manitol 10 g/L, agar 20 g/L), M3 [9] (yeast extract 5 g/L, dextrin 40 g/L, lactose 40 g/L, MOPS 20 g/L, ammonium acetate 1 g/L, agar 20 g/L), M4 [28] (yeast extract 1 g/L, beef extract 1 g/L, N-A amine (type A) 2 g/L, glucose 10 g/L, agar 20 g/L), M5 [13] yeast extract 4 g/L, malt extract 25 g/L, glucose 4 g/L, soy flour 6 g/L, agar 20 g/L) and M6 [29] (yeast extract 2 g/L, bacto-soytone 2 g/L, N-A amine (type A) 5 g/L, soluble starch 10 g/L, D-manitol 5 g/L, trace elements solution 1 ml/L, agar 20 g/L). Isatropolone A production level in these derivatives was determined by HPLC-MS as described above.

### Bioinformatics analysis

Protein functional categories were predicted using the XtalPred-RF tool and based on BLASTP results determined with the KEGG, COG, Swiss-Port, NT, and NR databases. The 3D structure of IsaS protein was simulated by online software Swiss Model [33] using the homologous modeling method. The consensus motif of IsaF binding sequence was obtained from the WebLogo website using MEME algorithm.

### Statistical analysis

All data for isatropolone production were presented as mean ± SEM. Statistical analyses involving comparison between groups of data were performed with Student’s *t*-test (two-tailed) as applicable by GraphPad Prism 7.0. Differences were considered statistically significant at *p < 0.05, and **p < 0.01.

### Electronic supplementary material

Below is the link to the electronic supplementary material.

Supplementary Material 1

## Data Availability

No datasets were generated or analysed during the current study.

## Abbreviations

ChIP: chromatin immunoprecipitation
EMSA: electrophoretic mobility shift assay
EIC: extracted ion chromatogram
HPLC: High-performance liquid chromatography
IPTG: isopropyl-β-thiogalactopyranoside
ISA: isatropolone
MS: Mass spectrometry
PBS: Phosphate-buffered saline
PCR: polymerase chain reaction
PKS: polyketide synthase
SARPs: *Streptomyces* antibiotic regulatory proteins
RT-qPCR: reverse transcription - quantitative PCR
