# Supplementary material for: Engineering Streptomyces sp. CPCC 204095 for the targeted high-level production of isatropolone A by elucidating its pathway-specific regulatory mechanism
Source: Microb Cell Fact. 2024 Apr 16;23:113. doi: 10.1186/s12934-024-02387-0 (PMC11020959; doi:10.1186/s12934-024-02387-0)
Supplement: Supplementary file 1 — Supplementary Material 1 [file 12934_2024_2387_MOESM1_ESM.docx]

**Supplementary material**

**Engineering *Streptomyces* sp. CPCC 204095 for the targeted high-level production of isatropolone A by elucidating its pathway-specific regulatory mechanism**

**Cong Zhang**^1,†^**, Qianqian Xu**^1,†^**, Jie Fu**^1^**, Linzhuan Wu**^1^**, Yihong Li**^1^**, Yuan Lu**^1^**, Yuanyuan Shi**^1^**, Hongmin Sun**^1^**, Xingxing Li**^1,^**^*^, Lifei Wang**^1,^**^*^, and Bin Hong**^1,^**^*^**

^1^CAMS Key Laboratory of Synthetic Biology for Drug Innovation, NHC Key Laboratory of Biotechnology for Microbial Drugs and State Key Laboratory of Bioactive Substances and Functions of Natural Medicines, Institute of Medicinal Biotechnology, Chinese Academy of Medical Sciences & Peking Union Medical College, Beijing, China.

^†^ Cong Zhang and Qianqian Xu contributed equally to this work.

**^*^ Correspondence:**

No. 1 Tiantan Xili, Institute of Medicinal Biotechnology, Chinese Academy of Medical Sciences & Peking Union Medical College, Beijing 100050, China.

Bin Hong, binhong69@hotmail.com, hongbin@imb.pumc.edu.cn;

Lifei Wang, lifeiwang2002@hotmail.com, wanglifei@imb.pumc.edu.cn;

Xingxing Li, xingxingsf@163.com, lixingxing@imb.pumc.edu.cn

**Table of Contents**

[Supplementary Methods 3](#_Toc162708575)

[1. The antibacterial activity assay. 3](#_Toc162708576)

[Supplementary Tables 4](#_Toc162708577)

[Table S1. Strains and plasmids used in this study. 4](#_Toc162708578)

[Table S2. The PCR primers used in this study. 7](#_Toc162708579)

[Table S3. Annotation and predicted function of proteins in *isa* cluster. 13](#_Toc162708580)

[Supplementary Figures 15](#_Toc162708581)

[Fig. S1. Sequence alignment of IsaJ and IsaF from *Streptomyces* sp. CPCC 204095 with homologous proteins. 15](#_Toc162708582)

[Fig. S2. Sequence alignment of IsaA from *Streptomyces* sp. CPCC 204095 with homologous proteins. 17](#_Toc162708583)

[Fig. S3. Sequence alignment of IsaB from *Streptomyces* sp. CPCC 204095 with homologous proteins. 18](#_Toc162708584)

[Fig. S4. Disruption of *isaF* and *isaJ* gene in *isa* biosynthetic gene cluster. 19](#_Toc162708585)

[Fig. S5. Disruption of *isaA* and *isaB* gene in *isa* biosynthetic gene cluster. 20](#_Toc162708586)

[Fig. S6. ChIP-PCR analysis of Flag-tagged IsaF. 21](#_Toc162708587)

[Fig. S7. EMSA analysis of the interaction of the promoter regions with puriﬁed IsaF. 22](#_Toc162708588)

[Fig. S8. HPLC analysis of the production of isatropolones in CKO. 23](#_Toc162708589)

[Fig. S9. ChIP-PCR analysis of Flag-tagged IsaJ. 24](#_Toc162708590)

[Fig. S10. EMSA analysis of the interaction of the promoter regions with puriﬁed IsaJ. 25](#_Toc162708591)

[Fig. S11. The sequence alignment of IsaS from *Streptomyces* sp. CPCC 204095 with other cytochrome P450s and predicted 3D structure of IsaS. 26](#_Toc162708592)

[Fig. S12. Disruption of *isaS* gene in *isa* biosynthetic gene cluster. 27](#_Toc162708593)

[Fig. S13. The antibacterial activity of isatropolones against *S. scabies*. 28](#_Toc162708594)

[Fig. S14. Construction of SKO derivatives with engineered *isaF*. 29](#_Toc162708595)

[Fig. S15. Isatropolone A production curves from SKO/kF with different inoculum spore concentrations. 30](#_Toc162708596)

[References 30](#_Toc162708597)

**Supplementary Methods**

**1. The antibacterial activity assay.**

The antibacterial activity of isatropolones against pathogenic *Streptomyces scabies* was performed by disk diffusion assay. *S. scabies* CGMCC 4.1765 was grown at 28 °C on mannitol soya flour (MS) agar [1] for sporulation. For antibacterial activity assay, 1 × 10^5^ spores per plate of *S. scabies* CGMCC 4.1765 were spread on MS plate. Individual ﬁlter paper disks containing different dose of puriﬁed isatropolone A (ISA A) or isatropolone C (ISA C) dissolved in methanol were applied to the *S. scabies* plate. 20 µL of methanol was used as negative control. The plates were then cultured at 28 °C for 3 days. After incubation, the inhibition zone diameter around the ﬁlter disks were recorded in millimeters (mm).

**Supplementary Tables**

**Table S1. Strains and plasmids used in this study.**

| **Strains and Plasmids** | **Description** | **Reference** |
| --- | --- | --- |
| **Strains** |  |  |
| *Streptomyces* sp. CPCC 204095 | Wild type (WT), isatropolones producer | [2] |
| *Streptomyces* sp. CPCC 204095/pL-isaF | *Streptomyces* sp. CPCC 204095 with the expression vector pL-isaF, Am^r^ | This study |
| *Streptomyces* sp. CPCC 204095/pL-isaJ | *Streptomyces* sp. CPCC 204095 with the expression vector pL-isaJ, Am^r^ | This study |
| *Streptomyces* sp. CPCC 204095/pL-isaS | *Streptomyces* sp. CPCC 204095 with the expression vector pL-isaS, Am^r^ | This study |
| *Streptomyces* sp. CPCC 204095/pL-isaA | *Streptomyces* sp. CPCC 204095 with the expression vector pL-isaA, Am^r^ | This study |
| *Streptomyces* sp. CPCC 204095/pL-isaB | *Streptomyces* sp. CPCC 204095 with the expression vector pL-isaB, Am^r^ | This study |
| *Streptomyces* sp. CPCC 204095/pSET152 | *Streptomyces* sp. CPCC 204095 with the control vector pSET152, Am^r^ | This study |
| *Streptomyces* sp. CPCC 204095/FKO | Mutant of *Streptomyces* sp. CPCC 204095 with disruption of *isaF* | This study |
| *Streptomyces* sp. CPCC 204095/JKO | Mutant of *Streptomyces* sp. CPCC 204095 with disruption of *isaJ* | This study |
| *Streptomyces* sp. CPCC 204095/SKO | Mutant of *Streptomyces* sp. CPCC 204095 with disruption of *isaS*, Tsr^r^ | This study |
| *Streptomyces* sp. CPCC 204095/AKO | Mutant of *Streptomyces* sp. CPCC 204095 with disruption of *isaA*, Tsr^r^ | This study |
| *Streptomyces* sp. CPCC 204095/BKO | Mutant of *Streptomyces* sp. CPCC 204095 with disruption of *isaB*, Tsr^r^ | This study |
| *Streptomyces* sp. CPCC 204095/CKO | Mutant of *Streptomyces* sp. CPCC 204095 with disruption of *isaC*, Tsr^r^ | This study |
| *Streptomyces* sp. CPCC 204095/FKO/pL-isaF | *Streptomyces* sp. CPCC 204095/FKO complemented with pL-isaF, Am^r^ | This study |
| *Streptomyces* sp. CPCC 204095/JKO/pL-isaJ | *Streptomyces* sp. CPCC 204095/JKO complemented with pL-isaJ, Am^r^ | This study |
| *Streptomyces* sp. CPCC 204095/SKO/pL-isaS | *Streptomyces* sp. CPCC 204095/SKO with the expression vector pL-isaS, Am^r^ | This study |
| *Streptomyces* sp. CPCC 204095/JKO/pL-isaS | *Streptomyces* sp. CPCC 204095/JKO with the expression vector pL-isaS, Am^r^ | This study |
| *Streptomyces* sp. CPCC 204095/FKO/pL-isaF-Flag | *Streptomyces* sp. CPCC 204095/FKO with the expression vector pL- isaF-Flag, Am^r^ | This study |
| *Streptomyces* sp. CPCC 204095/JKO/pL-isaJ-Flag | *Streptomyces* sp. CPCC 204095/JKO with the expression vector pL- isaJ-Flag, Am^r^ | This study |
| *Streptomyces* sp. CPCC 204095/FKO/pSET152 | *Streptomyces* sp. CPCC 204095/FKO with the vector pSET152, Am^r^ | This study |
| *Streptomyces* sp. CPCC 204095/JKO/pSET152 | *Streptomyces* sp. CPCC 204095/JKO with the vector pSET152, Am^r^ | This study |
| *Streptomyces* sp. CPCC 204095/SKO/pSET152 | *Streptomyces* sp. CPCC 204095/SKO with the vector pSET152, Am^r^ | This study |
| JKO/eF | *Streptomyces* sp. CPCC 204095/JKO with the vector pSET-eF, Am^r^ | This study |
| SKO/eF | *Streptomyces* sp. CPCC 204095/SKO with the vector pSET-eF, Am^r^ | This study |
| SKO/gF | *Streptomyces* sp. CPCC 204095/SKO with the vector pSET-gF, Am^r^ | This study |
| SKO/rF | *Streptomyces* sp. CPCC 204095/SKO with the vector pSET-rF, Am^r^ | This study |
| SKO/kF | *Streptomyces* sp. CPCC 204095/SKO with the vector pSET-kF, Am^r^ | This study |
| SKO/5768F | *Streptomyces* sp. CPCC 204095/SKO with the vector pSET-5768F, Am^r^ | This study |
| SKO/2027F | *Streptomyces* sp. CPCC 204095/SKO with the vector pSET-2027F, Am^r^ | This study |
| SKO/4727F | *Streptomyces* sp. CPCC 204095/SKO with the vector pSET-4727F, Am^r^ | This study |
| *Escherichia coli* DH5α | General cloning host | TransGen |
| *E. coli* DH5α/pET28a-SUMO-isaF | General cloning host of His-SUMO-isaF | This study |
| *E. coli* DH5α/pET28a-SUMO-isaJ | General cloning host of His-SUMO-isaJ | This study |
| *E. coli* ET12567/pUZ8002 | Strain used for *E. coli*-*Streptomyces* conjugation | [3] |
| *E. coli* BL21(DE3) | Strain used for the expression of IsaF and IsaJ protein | Novagen |
| *E. coli* BL21(DE3)/pET28a-SUMO-isaF | Strain used for the expression of His-SUMO-isaF | This study |
| *E. coli* BL21(DE3)/pET28a-SUMO-isaJ | train used for the expression of His-SUMO-isaJ | This study |
| *Streptomyces scabies* CGMCC 4.1765 | Strain used for isatropolones bioassays | CGMCC |
| **Plasmids** |  |  |
| pSET152 | *Streptomyces* integrative vector*, ϕC31*, *oriT*, Am^r^ | [1] |
| pICLSet | pSET152 derivative containing *ermE**p, Am^r^ | [4] |
| pL-isaF | pICLSet derivative plasmid containing complete coding region of *isaF*, Am^r^ | This study |
| pL-isaJ | pICLSet derivative plasmid containing complete coding region of *isaJ*, Am^r^ | This study |
| pL-isaS | pICLSet derivative plasmid containing complete coding region of *isaS*, Am^r^ | This study |
| pL-isaA | pICLSet derivative plasmid containing complete coding region of *isaA*, Am^r^ | This study |
| pL-isaB | pICLSet derivative plasmid containing complete coding region of *isaB*, Am^r^ | This study |
| pL-isaF-Flag | pICLSet derivative plasmid containing complete coding region of *isaF* with 3×Flag tag, Am^r^ | This study |
| pL-isaJ-Flag | pICLSet derivative plasmid containing complete coding region of *isaJ* with 3×Flag tag, Am^r^ | This study |
| pKC1139 | *E. coli-Streptomyces* shuttle vector, temperature sensitive replicon *pSG5*, Am^r^ | [5] |
| pKC-isaF | pKC1139 derivative plasmid containing homologous fragments flanking *isaF*, Am^r^ | This study |
| pKC-isaJ | pKC1139 derivative plasmid containing homologous fragments flanking *isaJ*, Am^r^ | This study |
| pKC-isaS | pKC1139 derivative plasmid containing homologous fragments flanking *isaS* and thiostrepton resistant gene, Tsr^r^, Am^r^ | This study |
| pKC-isaA | pKC1139 derivative plasmid containing homologous fragments flanking *isaA* and thiostrepton resistant gene, Tsr^r^, Am^r^ | This study |
| pKC-isaB | pKC1139 derivative plasmid containing homologous fragments flanking *isaB* and thiostrepton resistant gene, Tsr^r^, Am^r^ | This study |
| pKC-isaC | pKC1139 derivative plasmid containing homologous fragments flanking *isaC* and thiostrepton resistant gene, Tsr^r^, Am^r^ | This study |
| pET-28a(+) | *E. coli* expression vector, Km^r^ | Novagen |
| pET28a-SUMO-isaF | pET28a derivative plasmid containing His-SUMO fused IsaF, Km^r^ | This study |
| pET28a-SUMO-isaJ | pET28a derivative plasmid containing His-SUMO fused IsaJ, Km^r^ | This study |
| pSET-eF | A derivative of pSET152 containing the regulatory gene *isaF* driven by the *ermE**p promoter, Am^r^ | This study |
| pSET-gF | A derivative of pSET-eF with replacement of the *ermE**p promoter by the Pgapdh_(EL)_ promoter, Am^r^ | This study |
| pSET-rF | A derivative of pSET-eF with replacement of the *ermE**p promoter by the PrpsL_(XC)_ promoter, Am^r^ | This study |
| pSET-kF | A derivative of pSET-eF with replacement of the *ermE**p promoter by the *kasO**p promoter, Am^r^ | This study |
| pSET-5768F | A derivative of pSET-eF with replacement of the *ermE**p promoter by the Psco5768 promoter, Am^r^ | This study |
| pSET-2027F | A derivative of pSET-eF with replacement of the *ermE**p promoter by the Pisa2027 promoter, Am^r^ | This study |
| pSET-4727F | A derivative of pSET-eF with replacement of the *ermE**p promoter by the Pisa4727 promoter, Am^r^ | This study |

**Table S2. The PCR primers used in this study.**

| **Name** | **Sequence (5’-3’)** | **Purpose** |
| --- | --- | --- |
| **Gene expression** | | |
| isaF_F | TCCAAGGAGGACCCCACAATGTACTTCAAGGTGCTCGGC | Used for amplifying coding region of *isaF* |
| isaF_R | TGCAGGTCGACTCTAGAGGGTTCGTCGGGTTCAGGC |  |
| isaJ_F | TCCAAGGAGGACCCCACAGTGGGGACACGACTGAGG | Used for amplifying coding region of *isaJ* |
| isaJ_R | TGCAGGTCGACTCTAGAGCGGGATCGATTCGTAGGC |  |
| isaS_F | CCAAGGAGGACCCCACAATGTGCCTGGAACCGCAAAGGGAGT | Used for amplifying coding region of *isaS* |
| isaS_R | CCAAGGAGGACCCCACAATGTGCCTGGAACCGCAAAGGGAGT |  |
| isaA_F | TCCAAGGAGGACCCCACAATGGTCTGGCCGATCGAGG | Used for amplifying coding region of *isaA* |
| isaA_R | TGCAGGTCGACTCTAGAGCGGACAACTGGTGGTCCTGC |  |
| isaB_F | TCCAAGGAGGACCCCACAGTGTCTCCGCGTGGTGTGA | Used for amplifying coding region of *isaB* |
| isaB_R | TGCAGGTCGACTCTAGAGGCCATCGAGGCGTACGTATC |  |
| isaF-Flag_F | TCCAAGGAGGACCCCACAATGTACTTCAAGGTGCTCGGC | Used for amplifying coding region of *isaF* fused with 3×Flag tag |
| isaF-Flag_R | TGCAGGTCGACTCTAGAGCTTGTCGTCGTCGTCCTTGTAGTCGATGTCGTGGTCCTTGTAGTCGCCGTCGTGGTCCTTGTAGTCGGCGACGTGCCGGGACACG |  |
| isaJ-Flag_F | TCCAAGGAGGACCCCACAGTGGGGACACGACTGAGG | Used for amplifying coding region of *isaJ* fused with 3×Flag tag |
| isaJ-Flag_R | TGCAGGTCGACTCTAGAGCTTGTCGTCGTCGTCCTTGTAGTCGATGTCGTGGTCCTTGTAGTCGCCGTCGTGGTCCTTGTAGTCCGGGATCGATTCGTAGGC |  |
| isaF_F2 | actagtATGTACTTCAAGGTGCTCG | Used for amplifying coding region of *isaF* with *Spe*I and *Not*I sites |
| isaF_R2 | gcggccgcTCAGGCGACGTGCCGGG |  |
| ermE*p_F | tctagaGCGAGTGTCCGTTCGAGTG | Used for amplifying the region of *ermE**p promoter |
| ermE*p_R | actagtTGGGGTCCTCCTGTGGAGT |  |
| Pgapdh_(EL)__F | TAtctagaGCTGCTCCTTCGGTCGGACG | Used for amplifying the region of Pgapdh_(EL)_ promoter |
| Pgapdh_(EL)__R | TAactagtGCGTATCCCCTTTCAGATAC |  |
| PrpsL_(XC)__F | TAtctagaGCCCTGCAGGCGGAAGTCAG | Used for amplifying the region of PrpsL_(XC)_ promoter |
| PrpsL_(XC)__R | TAactagtTACGTCTCCGTCGTCT |  |
| kasO*p_F | TAtctagaTGTTCACATTCGAACGGTCT | Used for amplifying the region of *kasO**p promoter |
| kasO*p_R | TAactagtAACTCCCCCAGTCCTGCACG |  |
| Psco5768_F | TAtctagaCCCGAGGACTCGGGGCCGTAG | Used for amplifying the region of Psco5768 promoter |
| Psco5768_R | TAactagtCTCTCCGACCTCTCCCGGTG |  |
| Pisa2027_F | TAtctagaGCGCAACGCGGCCGGGGTCT | Used for amplifying the region of Pisa2027 promoter |
| Pisa2027_R | TAactagtAACCTCTCGGAACGATGGAAAC |  |
| Pisa4727_F | TAtctagaGCGCACCCCCTCGTGGCGGC | Used for amplifying the region of Pisa4727 promoter |
| Pisa4727_R | TAactagtGCGTGCTCCCTTGCTGAGT |  |
| **Gene mutation** | | |
| Tsr_F | TTCATATGGTCCGAGGAACAGAGGC | Used for amplifying coding region of tsr |
| Tsr_R | CCATATGTTGCGTTGGTGATTGC |  |
| isaFL_F | CCAAGCTTCGCAAGGAGCAGGACAGT | Used for amplifying 1,249 bp upstream arm of *isaF* |
| isaFL_R | TCCATATGCAGCAACAGCGACAGGGT |  |
| isaFR_F | TCCATATGCCCGATCTCGAACGCA | Used for amplifying 1,357 bp downstream arm of *isaF* |
| isaFR_R | GCTCTAGACGGAGATGACACCGTAGATG |  |
| isaJL_F | CCAAGCTTCTTCGAGTTCACCCACCG | Used for amplifying 1,077 bp upstream arm of *isaJ* |
| isaJL_R | TCCATATGCAGCAGCTTGCGTTCCTT |  |
| isaJR_F | GCCATATGCAGGACCTCCAGCGAAGA | Used for amplifying 1,017 bp downstream arm of *isaJ* |
| isaJR_R | GCTCTAGACCGCCGTGATGGATGAT |  |
| isaSL_F | CCAAGCTTGGCGACCATCATGTCCACG | Used for amplifying 1,503 bp upstream arm of *isaS* |
| isaSL_R | TCCATATGTACTCCCTTTGCGGTTCCAG |  |
| isaSR_F | TCCATATGCCTGCCGCTGAGCCGTACAT | Used for amplifying 1,197 bp downstream arm of *isaS* |
| isaSR_R | AGTCTAGACGACCACGCCGATGACTTCC |  |
| isaAL_F | CCAAGCTTACCGGGTCTTCCACAACATG | Used for amplifying 1309 bp upstream arm of *isaA* |
| isaAL_R | TCCATATGCATCGCCGCCTCCTCACT |  |
| isaAR_F | TCCATATGCATCGCCGCCTCCTCACT | Used for amplifying 1468 bp downstream arm of *isaA* |
| isaAR_R | GCTCTAGAAACGGGTGGACCTGGATGT |  |
| isaBL_F | CCAAGCTTCGGGGATCGTCAACACC | Used for amplifying 1416 bp upstream arm of *isaB* |
| isaBL_R | TCCATATGGGCGAAGAGCCGCTCAC |  |
| isaBR_F | GCCATATGAACCTGCTCGGCATCGC | Used for amplifying 1257 bp downstream arm of *isaB* |
| isaBR_R | GCTCTAGACGGCAGCTTCTACCGTCCT |  |
| isaCL_F | CCAAGCTTCGTCTCCCGCACGATCACCGAACT | Used for amplifying upstream arm of *isaC* |
| isaCL_R | TCCATATGCCACGCCTGGTCCTGATCCCTGTT |  |
| isaCR_F | TCCATATGTTCTCGCTGGCGGACC | Used for amplifying downstream arm of *isaC* |
| isaCR_R | GCTCTAGACTGCTGATGACCCTGTTCG |  |
| **Gene mutation analysis** | | |
| V_p152 | TTCGGCGGCTTCAAGTTCGG | Used for amplifying upstream and downstream region pSET152-attB integration site |
| V_attB | CGGTGGGGGTGCCAGGG |  |
| FKO-YP1 | CGCCGACTGGCACGAGAACA | Used for identifying *Streptomyces* sp. CPCC 204095/FKO |
| FKO-YP2 | CGACAGACCGCTCCACGATG |  |
| FKO-YP3 | GCGGGTCACGGCGTATCACA |  |
| FKO-YP4 | TCGCACTCCGGGTCAGGTTC |  |
| JKO-YP1 | ACACCGTACTCAGCGTGGGC | Used for identifying *Streptomyces* sp. CPCC 204095/JKO |
| JKO-YP2 | CAGACGAGTTGTCCCTTCTCCC |  |
| JKO-YP3 | GTCCTGCCACCGACGGTCAA |  |
| JKO-YP4 | GCGGAGCTACTAGGGCATTTCG |  |
| SKO-YP1 | ACAACTGGAGCGCCGGAACA | Used for identifying *Streptomyces* sp. CPCC 204095/SKO |
| SKO-YP2 | CGGACGGATTTGCGATGGTG |  |
| SKO-YP3 | CCGAGGTTACGTGTTCTCCCTTCC |  |
| SKO-YP4 | GATGAGCGGTCCCGAGTCCAA |  |
| SKO-YP5 | CGGCGAACTCCACAGCACGAT |  |
| SKO-YP6 | TGGTCAGGTCCGGCAACACC |  |
| AKO-YP1 | CTCCCTGAGCCTGGTCAACAC | Used for identifying *Streptomyces* sp. CPCC 204095/AKO |
| AKO-YP2 | GCCAAGACATTCGGCATCG |  |
| AKO-YP3 | CGTCGGTTCCAGCGACCACA |  |
| AKO-YP4 | TCCGCCTCGGTTTCCATCCC |  |
| AKO-YP5 | GGCAATCACCAACGCAAGG |  |
| AKO-YP6 | GGTCCGCCAGCGAGAACAT |  |
| BKO-YP1 | GGCAGGCATGTTCTGGCTCT | Used for identifying *Streptomyces* sp. CPCC 204095/BKO |
| BKO-YP2 | GGACGGATTTGCGATGGTGT |  |
| BKO-YP3 | GTCATCCGCCTCGGTTTCC |  |
| BKO-YP4 | CACGTCAAGTCGGTCCTGTCC |  |
| BKO-YP5 | CCTGGATGTCCGCCTGTTCG |  |
| BKO-YP6 | ATCGCGTCCTGGACCGTGTC |  |
| CKO-YP1 | GGACACTGCGGCACTACGA | Used for identifying *Streptomyces* sp. CPCC 204095/CKO |
| CKO-YP2 | GCGGCCAACCGATAAGC |  |
| CKO-YP3 | GGACGGATTTGCGATGGTGT |  |
| CKO-YP4 | CGTATAGGAGTGCCTGCGATGA |  |
| CKO-YP5 | GGCACTTGCCCATACCTTG |  |
| CKO-YP6 | TCGCTGGCGTTCATCCC |  |
| **RT-qPCR** | | |
| hrdB-RTF | CGTCGTTCGTCCACCACC | Used for detecting *hrdB* transcription |
| hrdB-RTR | CGTCCAGGTACATGCCGAC |  |
| isaA-RTF | GTGACGGCTCGGACACTG | Used for detecting *isaA* transcription |
| isaA-RTR | CCCGCAGCACGAGGAT |  |
| isaB-RTF | AGGTGCTGGAGCGGGACG | Used for detecting *isaB* transcription |
| isaB-RTR | GCGGAAATGGGCGTGCA |  |
| isaE-RTF | CCCATGTGACCGGTAGTGC | Used for detecting *isaE* transcription |
| isaE-RTR | GCGAGGTCCTCGTAACGC |  |
| isaF-RTF | ACCCTGTCGCTGTTGCTGC | Used for detecting *isaF* transcription |
| isaF-RTR | GCGTGGTCAGTGCGTTGG |  |
| isaG-RTF | CCGGCAGGTCGTGGAGAAGT | Used for detecting *isaG* transcription |
| isaG-RTR | TCCTGGATGCGGACGGTGA |  |
| isaH-RTF | GAAAGCGTTCTGGGACCTTCTGG | Used for detecting *isaH* transcription |
| isaH-RTR | AGCCCGTGCAGCTCGGGATT |  |
| isaJ-RTF | GAAAGCGTTCTGGGACCTTCTGG | Used for detecting *isaJ* transcription |
| isaJ-RTR | AGCCCGTGCAGCTCGGGATT |  |
| isaP-RTF | ATCAACTGCTGACCGTTCTCGC | Used for detecting *isaP* transcription |
| isaP-RTR | AGGGCGTCGAAGTCCGTGTC |  |
| isaS-RTF | CCGTTTGAGCGGACCACC | Used for detecting *isaS* transcription |
| isaS-RTR | TCGCCACCGAAGGGACAC |  |
| isaT-RTF | GGGTGAATCCGCTGGACTGG | Used for detecting *isaT* transcription |
| isaT-RTR | GACGACACCGGCGACATCA |  |
| **ChIP-PCR** | | |
| isaS-TChIP-F | GGCGAACTCCACAGCACGAT | Used for amplifying promoter region of *isaS* and *isaT* for ChIP-PCR |
| isaS-TChIP-R | TTGCGGTTCCAGGCACATT |  |
| isaRChIP-F | GACGGACGCAAGGATCGTG | Used for amplifying promoter region of *isaR* for ChIP-PCR |
| isaRChIP-R | TCGACCACGCCGATGACTT |  |
| isaLChIP-F | TCAGGTCCGGGCCGGGTGTT | Used for amplifying promoter region of *isaL* for ChIP-PCR |
| isaLChIP-R | CCGGATCTCGTGGGCTGAG |  |
| isaKChIP-F | GCGAACAGGTCACTTGCCG | Used for amplifying promoter region of *isaK* for ChIP-PCR |
| isaKChIP-R | CCGCCTACGAATCGATCCC |  |
| isaJChIP-F | GCGAGACACCGCCAGCCTG | Used for amplifying promoter region of *isaJ* for ChIP-PCR |
| isaJChIP-R | GCCGCCGTACGCCACCACA |  |
| isaHChIP-F | TGCCGGTGATGGCGACAGA | Used for amplifying promoter region of *isaH* for ChIP-PCR |
| isaHChIP-R | CGACGAACTCGACGAGTTGC |  |
| isaGChIP-F | CTGGAAGCACTTCTCCACGACC | Used for amplifying promoter region of *isaG* for ChIP-PCR |
| isaGChIP-R | GGCACGTCGCCTGAACCC |  |
| isaFChIP-F | GGCGTCAGATCCTTCGTACT | Used for amplifying promoter region of *isaF* for ChIP-PCR |
| isaFChIP-R | GGGTCACGGCGTATCACA |  |
| isaD-EChIP-F | GTCACATGGGCCATCGGG | Used for amplifying promoter region of *isaD* and *isaE* for ChIP-PCR |
| isaD-EChIP-R | CGTTCGGTGCGGCTGATGC |  |
| isaAChIP-F | GACATCCGGGCGACCTCCT | Used for amplifying promoter region of *isaA* for ChIP-PCR |
| isaAChIP-R | GTCCGGGTGCGGTAGGTAC |  |
| isaB-CChIP-F | GTCCTGATCCCTGTTGGCGATG | Used for amplifying promoter region of *isaB* and *isaC* for ChIP-PCR |
| IsaB-CChIP-R | ACACCACGCGGAGACACCGA |  |
| IsaU-VChIP-F | CCTCCAGCCAGGATCCAGC | Used for amplifying promoter region of *isaU* and *isaV* for ChIP-PCR |
| IsaU-VChIP-R | GTGTCCGGCGGATCCACTC |  |
| **Protein expression** | | |
| IsaJ-SUMO-GF | cacagagaacagattggtggatccGTGGGGACACGACTGAGGA | Used for amplifying His-SUMO-IsaJ |
| IsaJ-SUMO-GR | GTGCTCGAGTGCGGCCGCAAGCTTGCCGTCGAGTAGGTGCAGAA |  |
| IsaF-SUMO-GF | cacagagaacagattggtggatccATGTACTTCAAGGTGCTCGGC | Used for amplifying His-SUMO-IsaF |
| IsaF-SUMO-GR | GTGCTCGAGTGCGGCCGCAAGCTTGGTTCGTCGGGTTCAGGC |  |
| **EMSA** | | |
| isaD-Ep-EMSA-F | GAGGCACTACCGGTCACATG | Used for amplifying promoter region of *isaD-E* for EMSA |
| isaD-Ep-EMSA-R | TCATCGCAGGCACTCCTATAC |  |
| isaFp-EMSA-F | CGCAGCAGCAACAGCGACAG | Used for amplifying promoter region of *isaF* for EMSA |
| isaFp-EMSA-R | TGGTGAGCGACCTGGGCATC |  |
| isaGp-EMSA-F | ATTCGGTGAGGGTAAAGGC | Used for amplifying promoter region of *isaG* for EMSA |
| isaGp-EMSA-R | GATACTCGGCGACGGACTC |  |
| isaHp-EMSA-F | AACGCTTTCTTGCCGACC | Used for amplifying promoter region of *isaH* for EMSA |
| isaHp-EMSA-R | GGGCTTCGACTCGCTCATG |  |
| isaJp-EMSA-F | GCCAGCAGCTTGCGTTCC | Used for amplifying promoter region of *isaJ* for EMSA |
| isaJp-EMSA-R | GGCCACCACGGCTTCAACT |  |
| isaLp-EMSA-F | AGGTCCGGGCCGGGTGTT | Used for amplifying promoter region of *isaL* for EMSA |
| isaLp-EMSA-R | CGCAGGTACGGGAGGAGATC |  |
| isaPp-EMSA-F | AACGGTCAGCAGTTGATTCGG | Used for amplifying promoter region of *isaP* for EMSA |
| isaPp-EMSA-R | GGCGTCCACAAGGAGTTCTTCC |  |
| isaQp-EMSA-F | AGCAGCTTTCGCGTGACG | Used for amplifying promoter region of *isaQ* for EMSA |
| isaQp-EMSA-R | CGCTCGATTCCTTCCACG |  |
| isaRp-EMSA-F | GGGGACGGACGCAAGGAT | Used for amplifying promoter region of *isaR* for EMSA |
| isaRp-EMSA-R | CCTACCTGGTGACCTGGAACG |  |
| isaS-Tp-EMSA-F | CAGTCCAGCGGATTCACCC | Used for amplifying promoter region of *isaS-T* for EMSA |
| isaS-Tp-EMSA-F | TGCGGTTCCAGGCACATT |  |
| isaS-Tp-1-F | CAGTCCAGCGGATTCACCCC | Used for amplifying promoter region of *isaS-T*p-1 segment for EMSA |
| isaS-Tp-1-R | ACGGTAGTTGGCCGAACCTG |  |
| isaS-Tp-2-F | CCATCCAGGTTCGGCCAACT | Used for amplifying promoter region of *isaS-T*p-2 segment for EMSA |
| isaS-Tp-2-R | TGCGGTTCCAGGCACATTCC |  |
| isaU-Vp-EMSA-F | CGGGTCGGCGTAGATGGTGT | Used for amplifying promoter region of *isaU-V* for EMSA |
| isaU-Vp-EMSA-R | TCTCCTCCGCGTTCGTGTCC |  |
| isaWp-EMSA-F | GCAGAACTGATTCGACGAGC | Used for amplifying promoter region of *isaW* for EMSA |
| isaWp-EMSA-R | TGGACGGCGATCACTACG |  |

**Table S3. Annotation and predicted function of proteins in *isa* cluster.**

| **Name** | **Siza (aa)** | **Proposed Function** | **BLAST Closest Homolog (identity %)** |
| --- | --- | --- | --- |
| IsaA | 253 | MerR regulator | 97 [IstA, *Streptomyces* Gö66] |
| IsaB | 204 | TetR regulator | 97 [IstB, *Streptomyces* Gö66] |
| IsaC | 281 | Methyltransferase | 98 [IstC, *Streptomyces* Gö66] |
| IsaD | 493 | Drug resistance transporter | 97 [IstD, *Streptomyces* Gö66] |
| IsaE | 526 | FAD-linked oxidase | 99 [IstE, *Streptomyces* Gö66] |
| IsaF | 296 | SARP regulator | 98 [IstF, *Streptomyces* Gö66] |
| IsaG | 84 | Acyl carrier protein | 94 [IstG, *Streptomyces* Gö66] |
| IsaH | 419 | Beta-ketosynthase | 99 [IstH, *Streptomyces* Gö66] |
| IsaI | 436 | Chain length factor | 99 [IstI, *Streptomyces* Gö66] |
| IsaJ | 263 | SARP family regulator | 98 [IstJ, *Streptomyces* Gö66] |
| IsaK | 385 | Glycosyl transferase | 99 [IstK, *Streptomyces* Gö66] |
| IsaL | 370 | Dehydrogenase | 97 [IstL, *Streptomyces* Gö66] |
| IsaM | 276 | Cyclase/aromatase | 99 [IstM, *Streptomyces* Gö66] |
| IsaN | 249 | Cyclase | 99 [IstN, *Streptomyces* Gö66] |
| IsaO | 199 | Dihydropteridine reductase | 99 [IstO, *Streptomyces* Gö66] |
| IsaP | 597 | Oxygenase | 99 [IstP, *Streptomyce*s Gö66] |
| IsaQ | 280 | NAD dependent epimerase/dehydratase | 98 [IstQ, *Streptomyces* Gö66] |
| IsaR | 330 | 3-oxoacyl-ACP synthase III | 99 [IstR, *Streptomyces* Gö66] |
| IsaS | 409 | P450 monooxygenase | 99 [IstS, *Streptomyces* Gö66] |
| IsaT | 308 | Alcohol dehydrogenase | 99 [IstT, *Streptomyces* Gö66] |
| IsaU | 357 | Malonyl-CoA acyltransferase | 97 [IstU, *Streptomyces* Gö66] |
| IsaV | 246 | Methyltransferase | 63 [*Streptomyces* yaneogriseus] |
| IsaW | 390 | Methyltransferase | 37 [*Streptomyces* sp. MK730-62F2] |
| Isa1' | 678 | Glycosyltransferase | 95 [*Streptomyces* *atratus*] |
| Isa2' | 199 | dTDP-4-dehydrorhamnose 3,5-epimerase | 97 [*Streptomyces* sp. Ncost-T10-10d] |
| Isa3' | 273 | Methyltransferase | 98 [*Streptomyces* *atratus*] |
| Isa4' | 294 | dTDP-4-dehydrorhamnose reductase | 98 [*Streptomyces atratus*] |
| Isa5' | 328 | dTDP-glucose 4,6-dehydratase | 99 [*Streptomyces* sp. DvalAA-43] |
| Isa6' | 361 | Glucose-1-phosphate thymidylyltransferase | 98 [*Streptomyces* *atratus*] |
| Isa7' | 400 | Glycosyltransferase family protein | 97 [*Streptomyces* *atratus*] |

**Supplementary Figures**


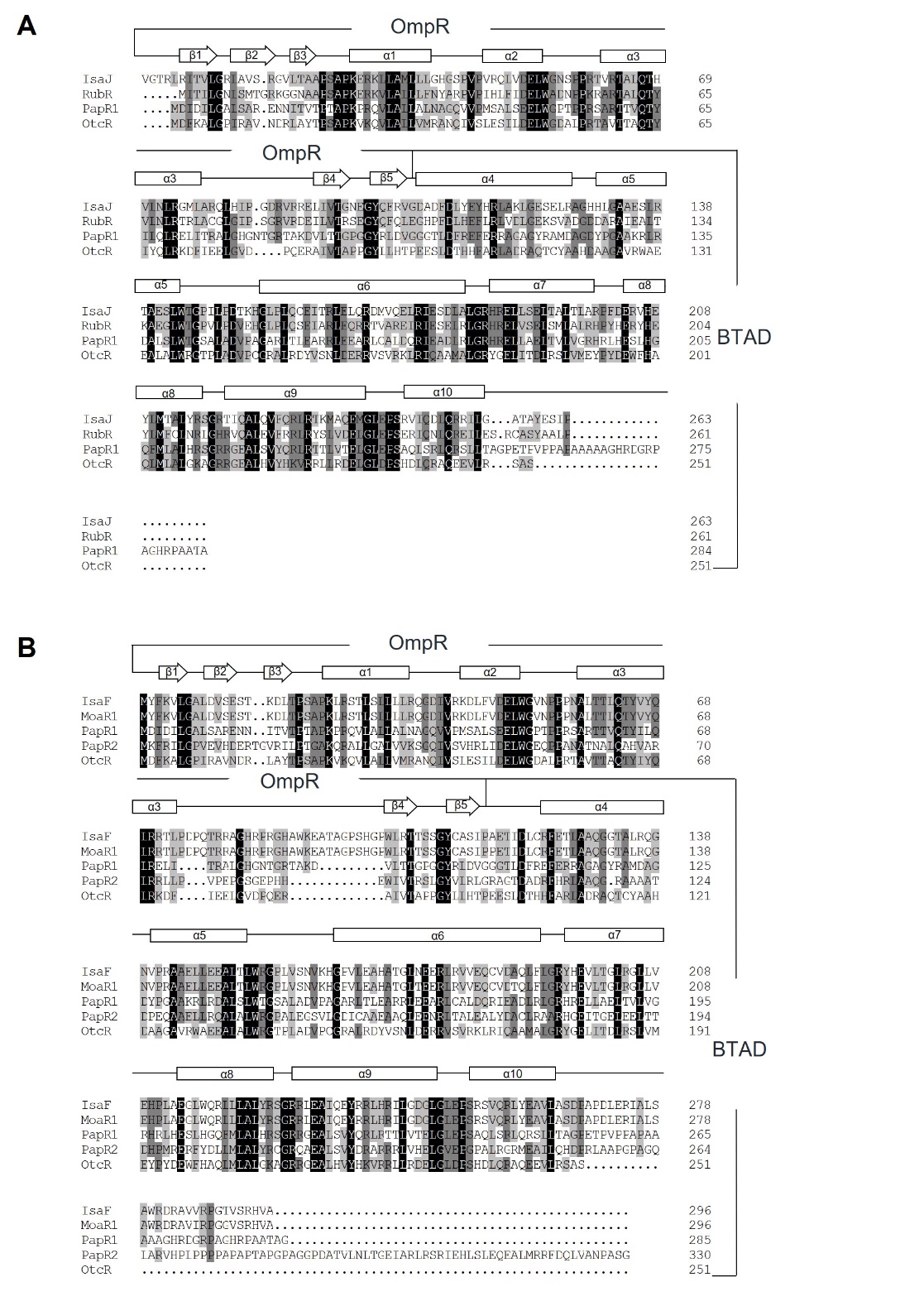


**Fig. S1. Sequence alignment of IsaJ and IsaF from *Streptomyces* sp. CPCC 204095 with homologous proteins.**

(**A**) Amino acid sequence multi-alignment of IsaJ with SARP family proteins RubR of *Streptomyces* sp. KIB-H033[6], PapR1 of *S. pristinaespiralis* [7] and OtcR of *S. rimosus* [8]. (**B**) Amino acid sequence multi-alignment of IsaF with SARP family proteins *Streptomyces* sp. ADI95-17 MoaR1 (GenBank: RPK72961.1), *S. pristinaespiralis* PapR1 [7], *S. pristinaespiralis* PapR2 [7] and *S. rimosus* OtcR [8] The horizontal line above the sequences identifies the predicted domains in IsaJ and IsaF, including the N-terminus OmpR-type DBD domain and the C-terminus BTAD domain, the arrow indicates the *β*-sheet region, the box indicates the *α*-helix region. Identical residues were shaded in black, while similar residues were shaded in gray.


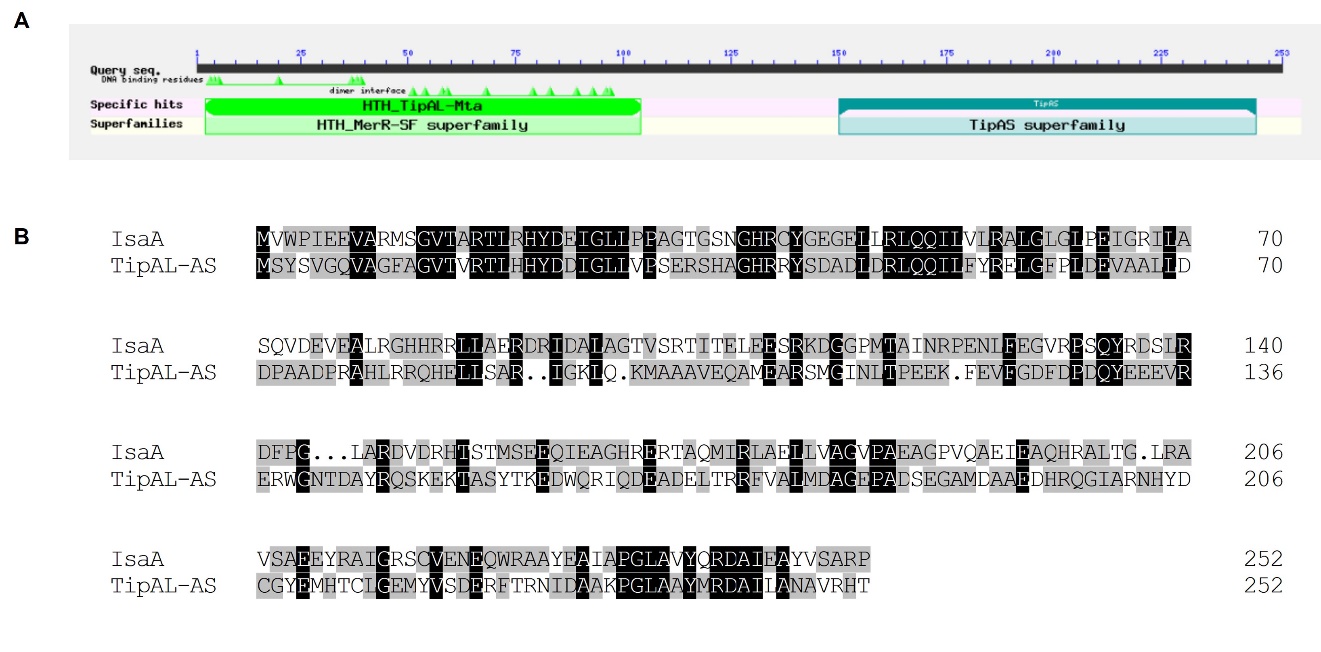


**Fig. S2. Sequence alignment of IsaA from *Streptomyces* sp. CPCC 204095 with homologous proteins.**

(**A**) Predicted MerR-like HTH DNA binding domain and a C-terminal domain of the TipAS family in IsaA. (**B**) Amino acid sequence alignment of IsaA with MerR family protein TipAL-AS of *S. lividans* [9]. Identical residues were shaded in black, while similar residues were shaded in gray.


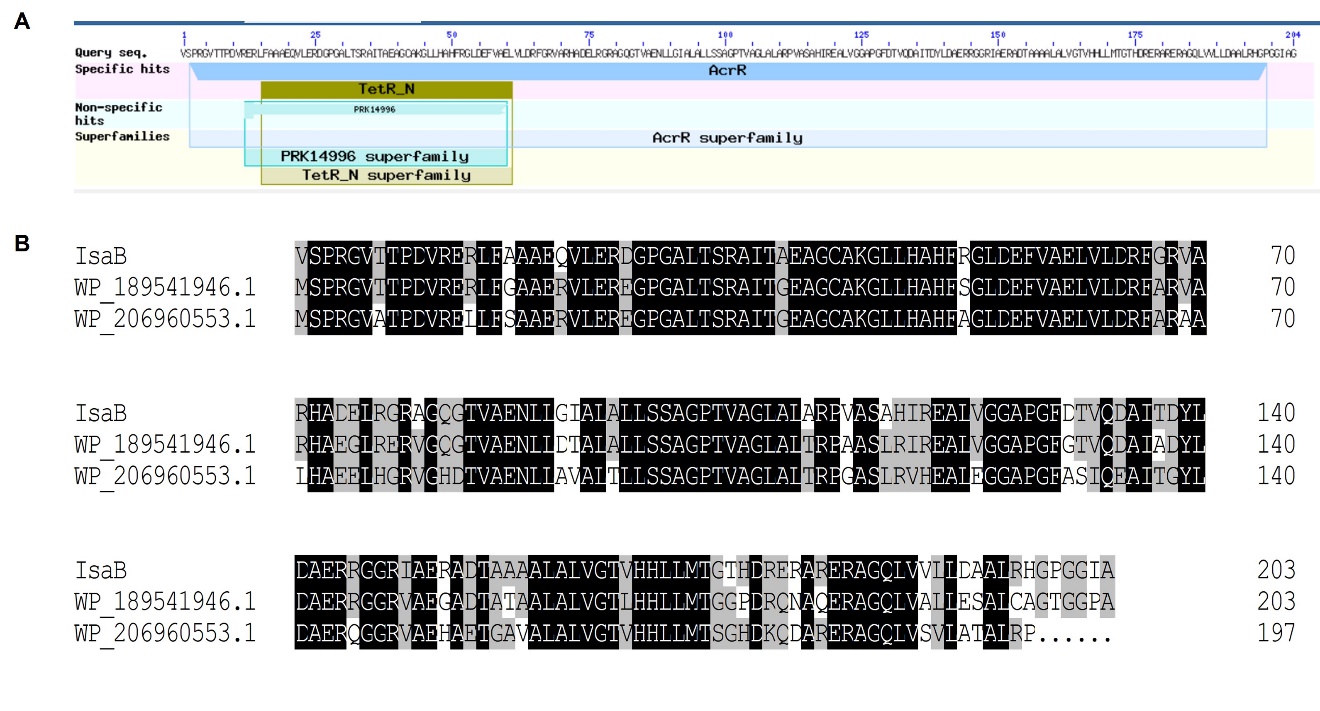


**Fig. S3. Sequence alignment of IsaB from *Streptomyces* sp. CPCC 204095 with homologous proteins.**

(**A**) Predicted HTH DNA binding domain of TetR family in IsaB. (**B**) Amino acid sequence multi-alignment of IsaB with TetR family ptoteins WP_189541946.1 of *S. gelaticus* and WP_206960553.1 of *S. beijiangensis*. Identical residues were shaded in black, while similar residues were shaded in gray.


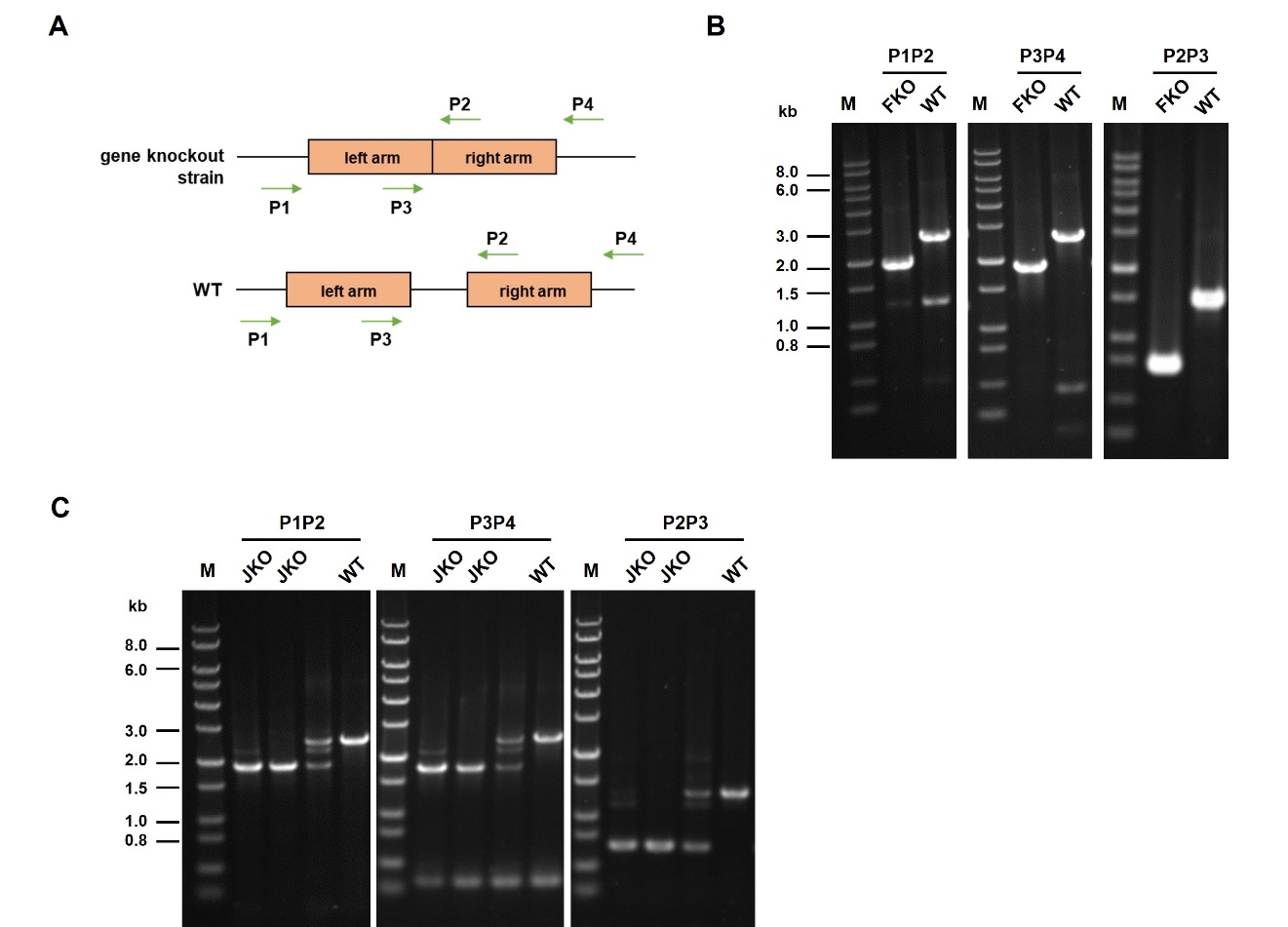


**Fig. S4. Disruption of *isaF* and *isaJ* gene in *isa* biosynthetic gene cluster.**

(**A**) Schematic representation of primers designed for knockout strain verification. (**B**) PCR analysis of the *isaF* knockout mutant (FKO), and the wild type strain (WT, *Streptomyces* sp. CPCC 204095) were carried out with primers listed in Table S2. (**C**) PCR analysis of the *isaJ* knockout mutants (JKO), and the wild type strain (WT, *Streptomyces* sp. CPCC 204095) were carried out with primers listed in Table S2. M, DNA molecular ladder.


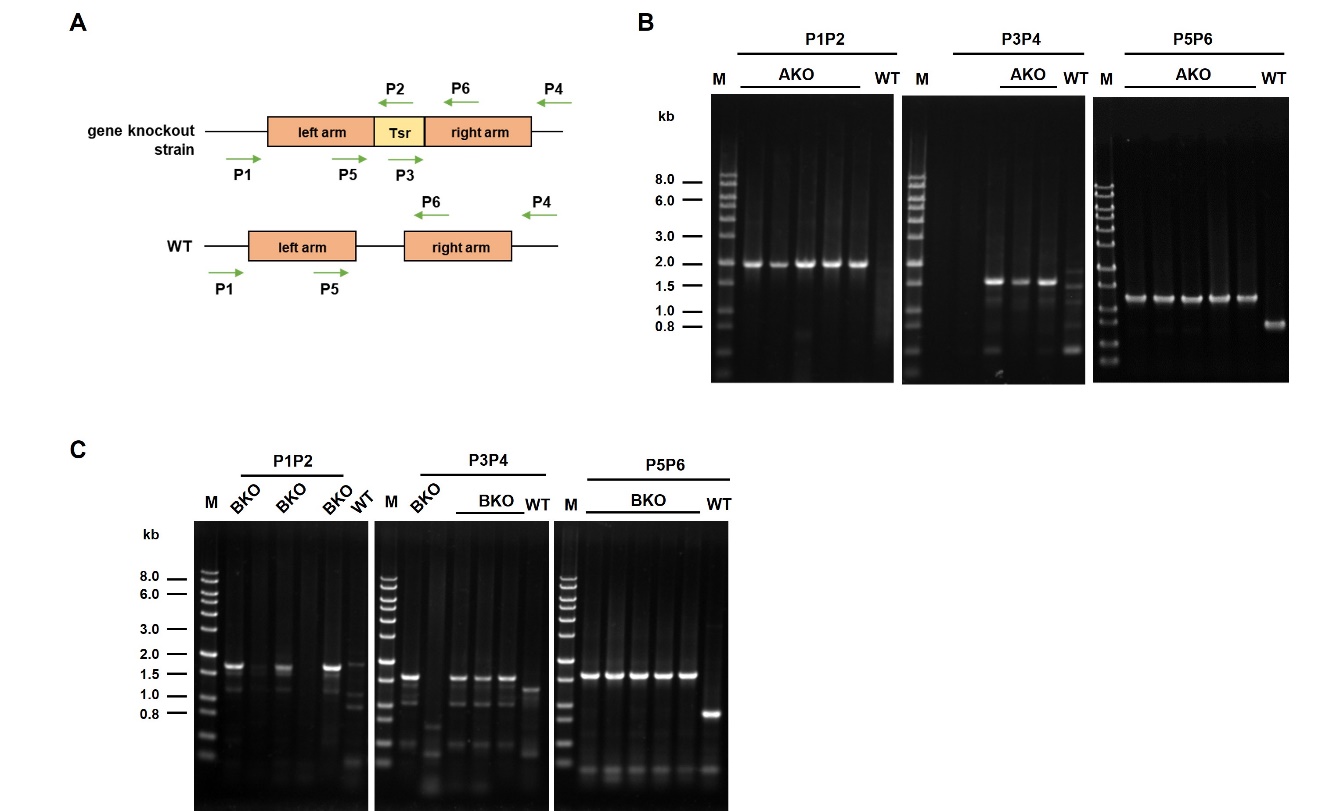


**Fig. S5. Disruption of *isaA* and *isaB* gene in *isa* biosynthetic gene cluster.**

(**A**) Schematic representation of primers designed for knockout strain verification. (**B**) PCR analysis of the *isaA* knockout mutants (AKO), and the wild type strain (WT, *Streptomyces* sp. CPCC 204095) were carried out with primers listed in Table S2. (**C**) PCR analysis of the *isaB* knockout mutants (BKO), and the wild type strain (WT, *Streptomyces* sp. CPCC 204095) were carried out with primers listed in Table S2. M, DNA molecular ladder.


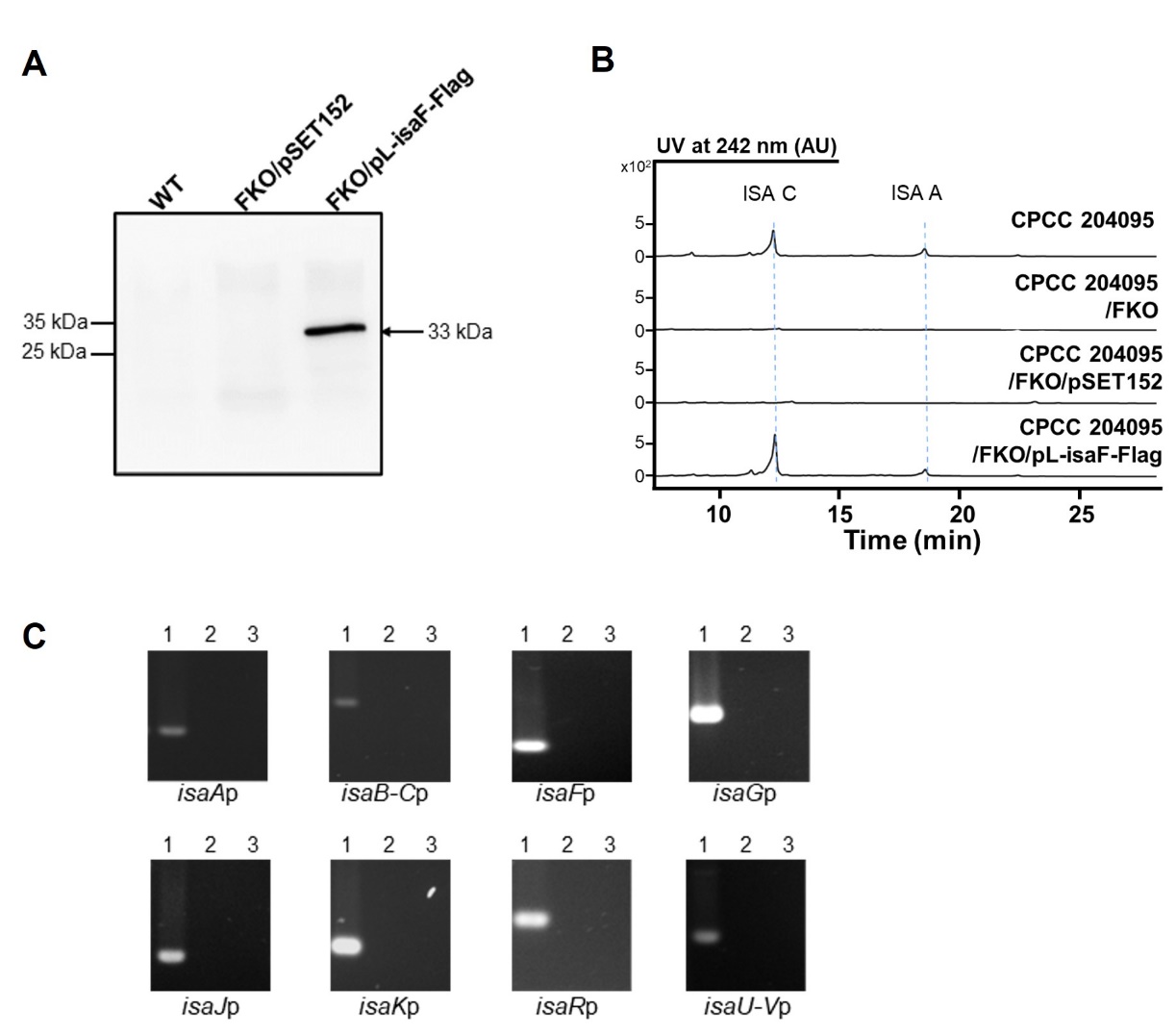


**Fig. S6.** **ChIP-PCR analysis of Flag-tagged IsaF.**

(**A**) Western blot analysis of the IsaF-flag fused protein in complement strain (FKO/pL-isaF-Flag) and the empty vector control strain (FKO/pSET152). The mycelia of strain were collected for the extraction of total protein at 48 h of the fermentation on ISP2 agar at 28 °C. Anti-Flag antibody was used as primary antibody. (**B**) HPLC analysis of isatropolones produced by the wild type strain (CPCC 204095), the *isaF* knockout strain (FKO), the empty vector control strain (FKO/pSET152) and flag-tagged IsaF complement strain (FKO/pL-isaF-Flag). (**C**) PCR using primers ﬂanking target promoter regions were performed with immunoprecipitated DNA from FKO/pL-isaF-Flag (lanes 1–3). Total DNA prior to immunoprecipitation (lane 1) was used as positive control for PCR. DNA treated with anti-Flag antibody (lane 2) or with IgG antibody (lane 3) was analyzed by PCR.


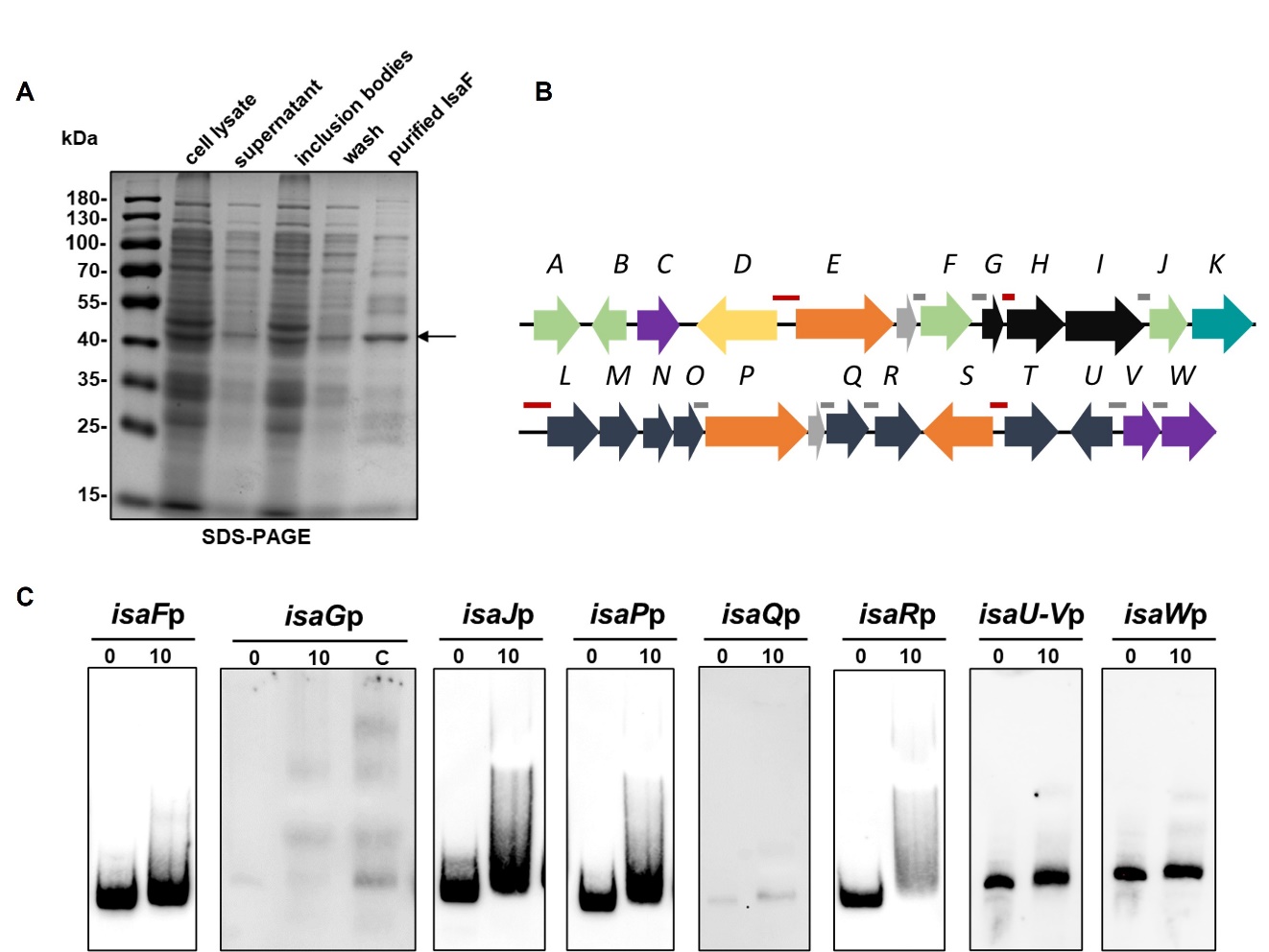


**Fig. S7. EMSA analysis of the interaction of the promoter regions with puriﬁed IsaF.**

(**A**) SDS-PAGE analysis of His-SUMO-tagged IsaF. The recombinant His-SUMO-tagged IsaF was purified and visualized by Coomassie Brilliant Blue staining after SDS-PAGE on 10 % polyacrylamide gel. The theoretical molecular weight of His-SUMO-tagged IsaF is 47 kDa. (**B**) Schematic representation of probe designed for EMSA. The red bar represents the position that IsaF can bind. The gray bars represent the regions that IsaF can not bind. (**C**) His-SUMO-tagged IsaF could not bind to the promoter regions of these 8 genes identified by EMSA. 10 µl His-SUMO-tagged IsaF was incubated with the probe. C, 10 µl His-SUMO-tagged IsaF was incubated with 200-fold excess unlabeled speciﬁc competitor.


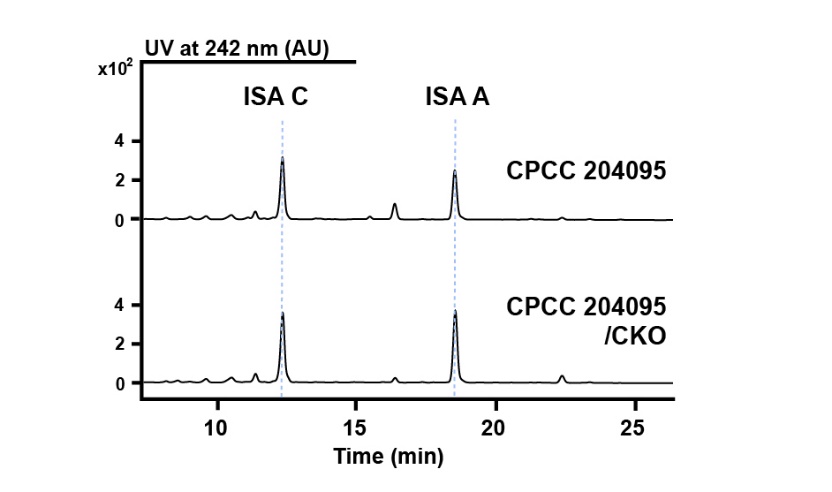


**Fig. S8. HPLC analysis of the production of isatropolones in CKO.**

HPLC analyses monitor the production of isatropolone A (ISA A) and isatropolone C (ISA C) in the wild type strain (CPCC 204095), and the *isaC* gene knockout strain (CKO).


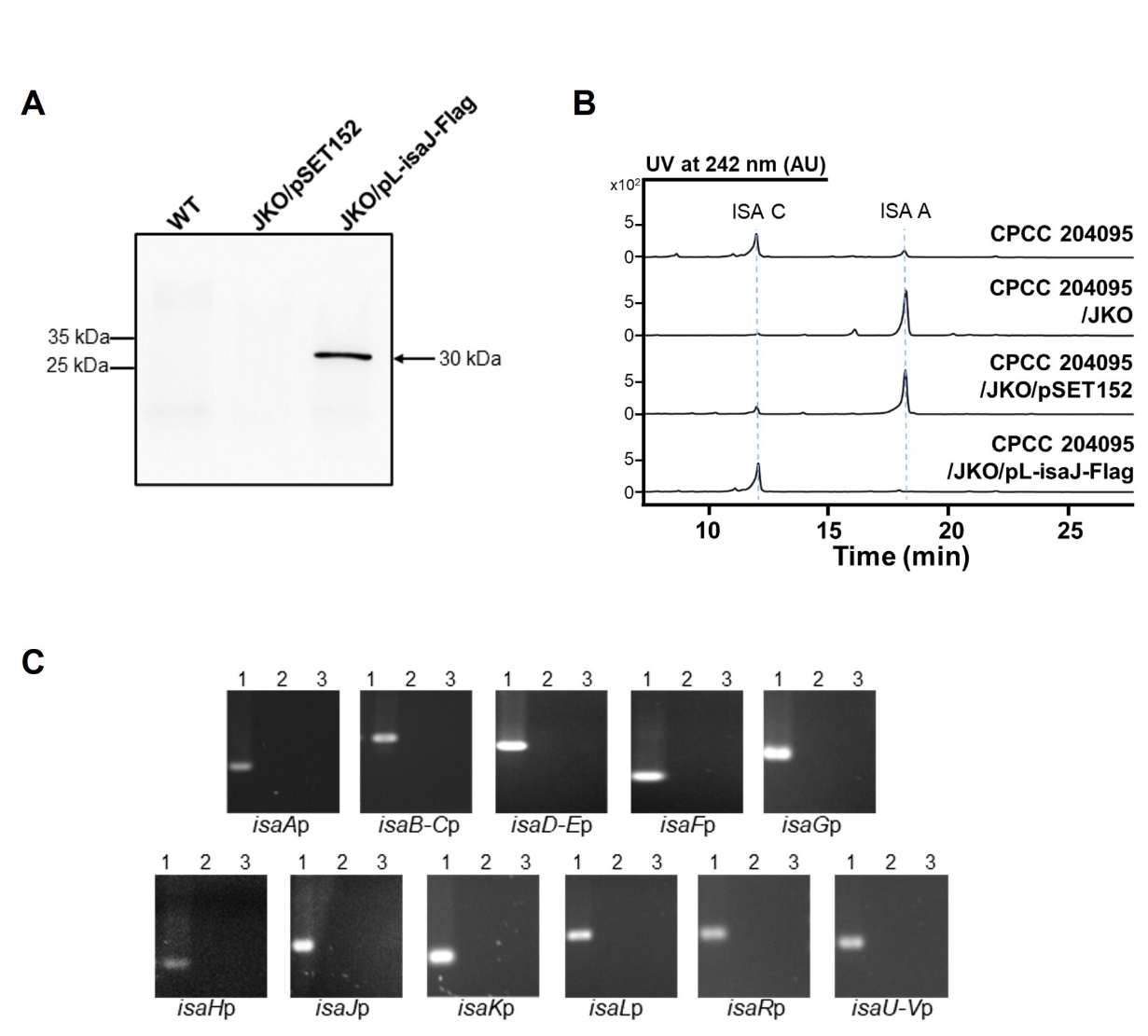


**Fig. S9. ChIP-PCR analysis of Flag-tagged IsaJ.**

(**A**) Western blot analysis of the IsaJ-flag fused protein in complement strain (JKO/pL-isaJ-Flag) and the empty vector control strain (JKO/pSET152). The mycelia of strain were collected for the extraction of total protein at 48 h of the fermentation on ISP2 agar at 28 °C. Anti-Flag antibody was used as primary antibody. (**B**) HPLC analysis of isatropolones produced by the wild type strain (CPCC 204095), the *isaJ* knockout strain (JKO), the empty vector control strain (JKO/pSET152) and flag-tagged IsaJ complement strain (JKO/pL-isaJ-Flag). (**C**) PCR using primers ﬂanking target promoter regions were performed with immunoprecipitated DNA from JKO/pL-isaJ-Flag (lanes 1–3). Total DNA prior to immunoprecipitation (lane 1) was used as positive control for PCR. DNA treated with anti-Flag antibody (lane 2) or with IgG antibody (lane 3) was analyzed by PCR.


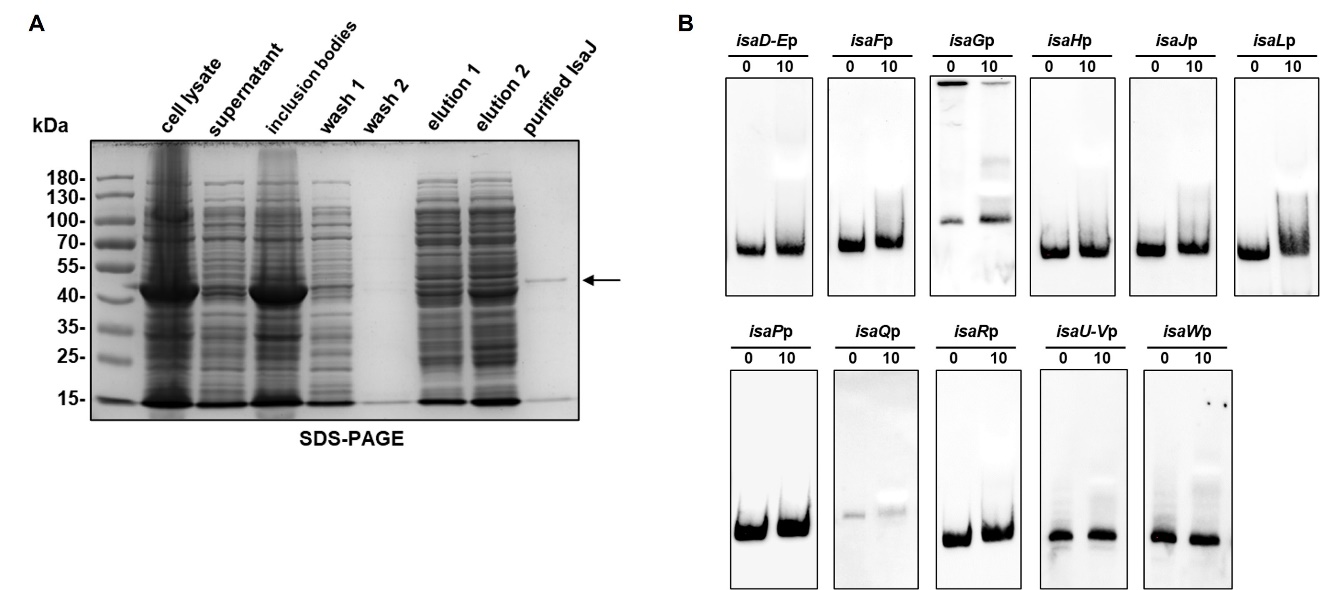


**Fig. S10. EMSA analysis of the interaction of the promoter regions with puriﬁed IsaJ.**

(**A**) SDS-PAGE analysis of His-SUMO-tagged IsaJ. The recombinant His-SUMO-tagged IsaJ was purified and visualized by Coomassie Brilliant Blue staining after SDS-PAGE on 10 % polyacrylamide gel. The theoretical molecular weight of His-SUMO-tagged IsaJ is 44 kDa. (**B**) His-SUMO-tagged IsaJ could not bind to the promoter regions of these 11 genes identified by EMSA. 10 µl His-SUMO-tagged IsaJ was incubated with the probe.


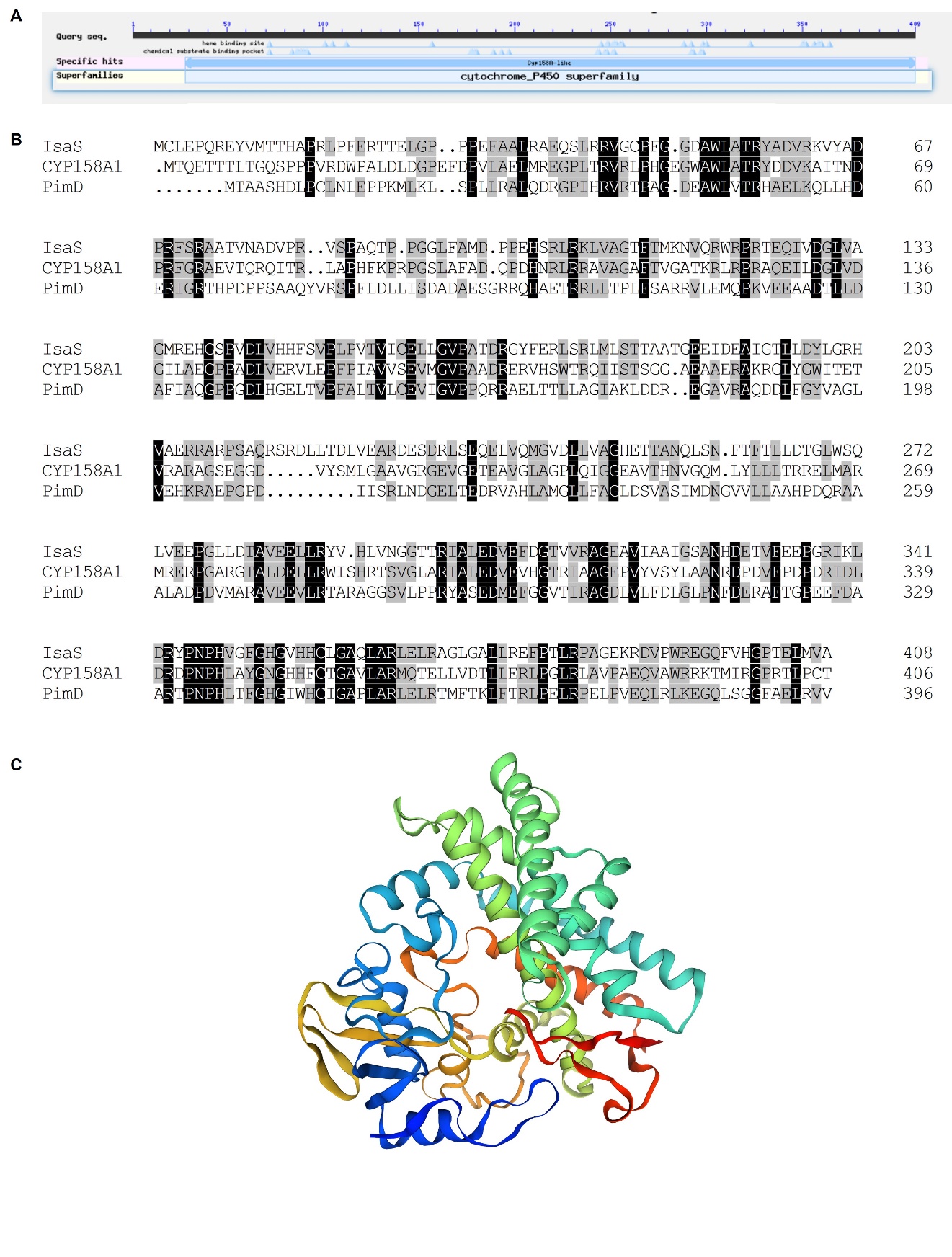


**Fig. S11. The sequence alignment of IsaS from *Streptomyces* sp. CPCC 204095 with other cytochrome P450s and** **predicted 3D structure of IsaS.**

(**A**) Predicted domains in IsaS. (**B**) Amino acid sequence multi-alignment of IsaS with cytochrome P450s CYP158A1 of *S. coelicolor* [10] and PimD of *S. natalensis* [11]*.* Identical residues were shaded in black, while similar residues were shaded in gray. (**C**) Predicted 3D structure of IsaS. Helices and strands are color-labelled.


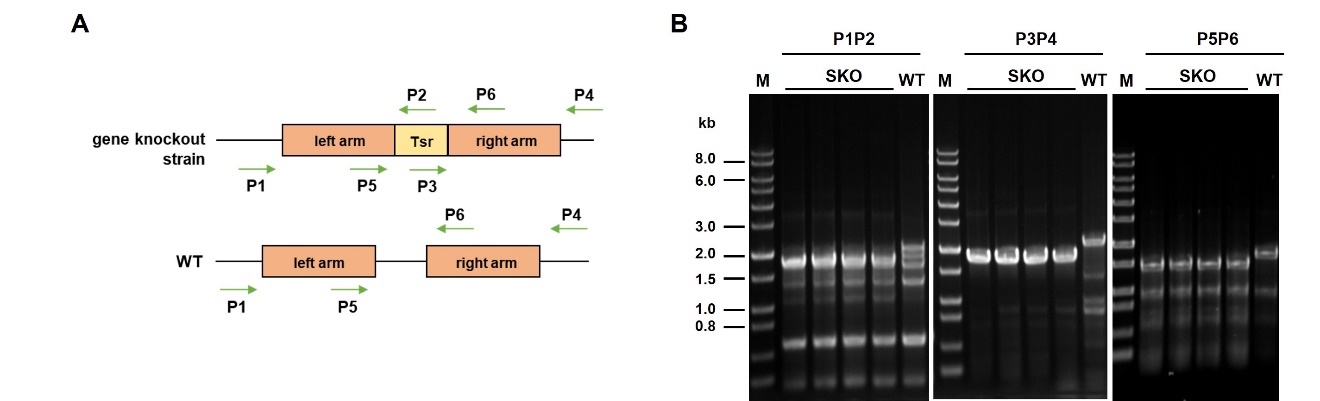


**Fig. S12. Disruption of *isaS* gene in *isa* biosynthetic gene cluster.**

(**A**) Schematic representation of primers designed for knockout strain verification. (**B**) PCR analysis of the *isaS* knockout mutants (SKO), and the wild type strain (WT, *Streptomyces* sp. CPCC 204095) were carried out with primers listed in Table S2. M, DNA molecular ladder.


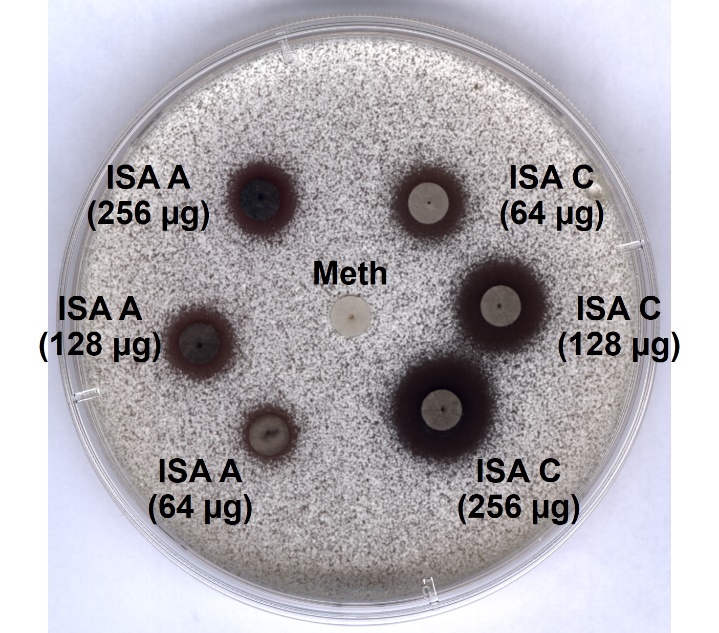


|  | Zone of inhibition (mm) against *Streptomyces scabies* | | |
| --- | --- | --- | --- |
|  | 42 h | 48 h | 72 h |
| ISA C (256 µg) | 18 | 16 | 13 |
| ISA C (128 µg) | 15 | 14 | 10 |
| ISA C (64 µg) | 12 | 11 | 9 |
| ISA A (256 µg) | 13 | 12 | 9 |
| ISA A (128 µg) | 12 | 11 | 8 |
| ISA A (64 µg) | 10 | 8 | 0 |
| Meth (20 µl) | 0 | 0 | 0 |

**Fig. S13. The antibacterial activity of isatropolones against *S. scabies*.**

The antibacterial activity of isatropolones against pathogenic *S. scabies* was performed by disk diffusion assay. *S. scabies* CGMCC 4.1765 was grown on a solid MS plate in the presence of ﬁlter paper disks containing different dose of puriﬁed isatropolone A (ISA A) or isatropolone C (ISA C) dissolved in methanol. Meth, methanol only. The inhibition zone diameter around the ﬁlter disks were recorded in millimeters (mm). The figure showed the result of 48 h of cultivation at 28 °C.


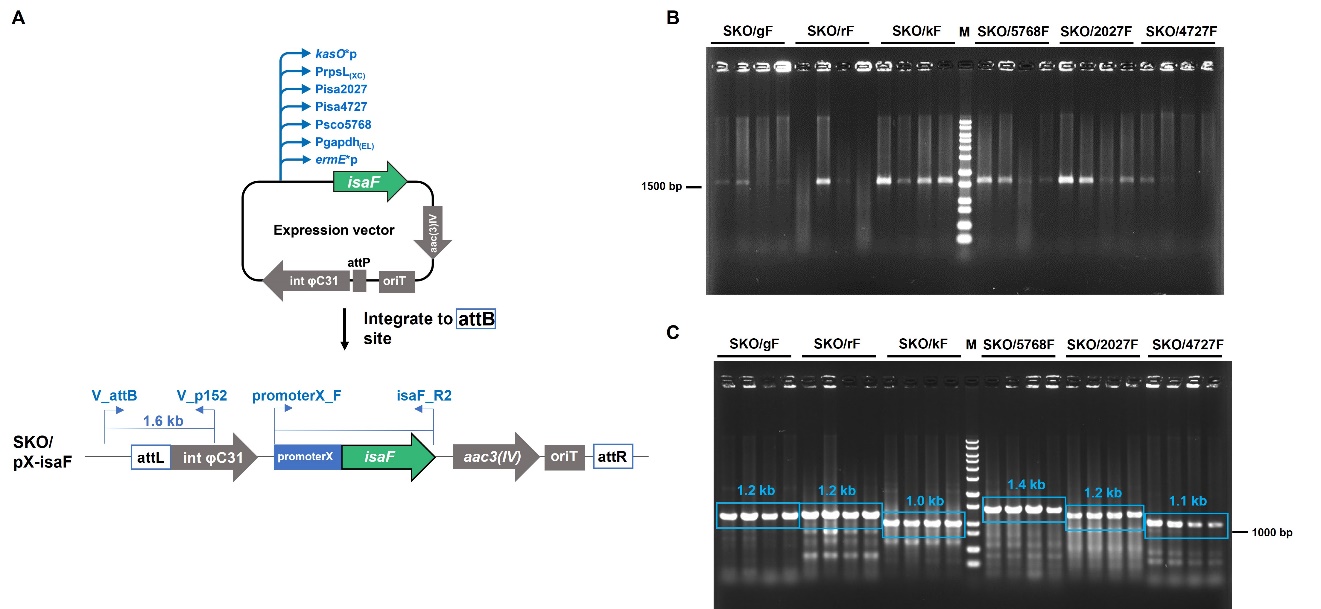


**Fig. S14. Construction of SKO derivatives with engineered *isaF*.**

(**A**) Schematic representation of primers designed for engineered strain verification. (**B, C**) PCR analysis of the construction of SKO derivatives with different promoters driving the expression of *isaF* were carried out with two pairs of primers (V_attB and V_p152 (B), promoter X_F and isaF_R2 (C)) listed in Table S2. M, DNA molecular ladder. The size of the predicted and observed PCR fragments was shown by rectangular box.


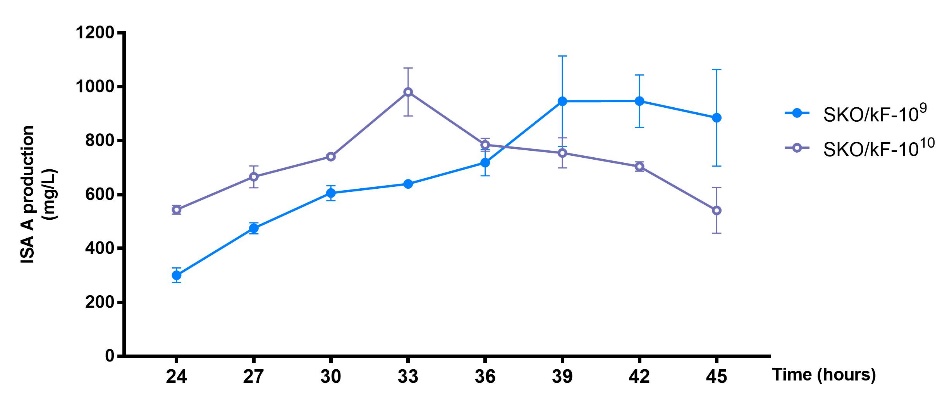


**Fig. S15. Isatropolone A production curves from SKO/kF with different inoculum spore concentrations.**

The production curves of isatropolone A were obtained from SKO/kF on M5 agar medium with different inoculum spore concentrations (10^9^ or 10^10^ spores/plate). Values are presented as mean ± SEM (two independent conjugants).

**References**

1. Kieser T, Bibb MJ, Chater KF, Butter M, Hopwood D, Bittner ML, Buttner MJ: Practical *streptomyces* genetics: a laboratory manual. 2000.

2. Li L, Li S, Jiang B, Zhang M, Zhang J, Yang B, Li L, Yu L, Liu H, You X, et al: Isarubrolones containing a pyridooxazinium unit from *Streptomyces* as autophagy activators. *J Nat Prod* 2019, 82:1149-1154.

3. Paget MS, Chamberlin L, Atrih A, Foster SJ, Buttner MJ: Evidence that the extracytoplasmic function sigma factor sigmaE is required for normal cell wall structure in *Streptomyces coelicolor* A3(2). *J Bacteriol* 1999, 181:204-211.

4. Hong B, Phornphisutthimas S, Tilley E, Baumberg S, McDowall KJ: Streptomycin production by *Streptomyces* *griseus* can be modulated by a mechanism not associated with change in the adpA component of the A-factor cascade. *Biotechnol Lett* 2007, 29:57-64.

5. Bierman M, Logan R, O'Brien K, Seno ET, Rao RN, Schoner BE: Plasmid cloning vectors for the conjugal transfer of DNA from *Escherichia coli* to *Streptomyces* spp. *Gene* 1992, 116:43-49.

6. Yan Y, Yang J, Yu Z, Yu M, Ma YT, Wang L, Su C, Luo J, Horsman GP, Huang SX: Non-enzymatic pyridine ring formation in the biosynthesis of the rubrolone tropolone alkaloids. *Nat Commun* 2016, 7:13083.

7. Mast Y, Guezguez J, Handel F, Schinko E: A complex signaling cascade governs pristinamycin biosynthesis in *Streptomyces pristinaespiralis*. *Appl Environ Microbiol* 2015, 81:6621-6636.

8. Yin S, Wang W, Wang X, Zhu Y, Jia X, Li S, Yuan F, Zhang Y, Yang K: Identification of a cluster-situated activator of oxytetracycline biosynthesis and manipulation of its expression for improved oxytetracycline production in *Streptomyces rimosus*. *Microb Cell Fact* 2015, 14:46.

9. Kahmann JD, Sass HJ, Allan MG, Seto H, Thompson CJ, Grzesiek S: Structural basis for antibiotic recognition by the TipA class of multidrug-resistance transcriptional regulators. *Embo j* 2003, 22:1824-1834.

10. Zhao B, Lamb DC, Lei L, Kelly SL, Yuan H, Hachey DL, Waterman MR: Different binding modes of two flaviolin substrate molecules in cytochrome P450 158A1 (CYP158A1) compared to CYP158A2. *Biochemistry* 2007, 46:8725-8733.

11. Mendes MV, Recio E, Fouces R, Luiten R, Martín JF, Aparicio JF: Engineered biosynthesis of novel polyenes: a pimaricin derivative produced by targeted gene disruption in *Streptomyces natalensis*. *Chem Biol* 2001, 8:635-644.
